# Triple-negative breast cancer modifies the systemic immune landscape and alters neutrophil functionality

**DOI:** 10.1038/s41523-025-00721-2

**Published:** 2025-01-23

**Authors:** Noor A. M. Bakker, Hannah Garner, Ewald van Dyk, Elisa Champanhet, Chris Klaver, Maxime Duijst, Leonie Voorwerk, Iris Nederlof, Rosie Voorthuis, Marte C. Liefaard, Marja Nieuwland, Iris de Rink, Onno B. Bleijerveld, Hendrika M. Oosterkamp, Lodewyk F. A. Wessels, Marleen Kok, Karin E. de Visser

**Affiliations:** 1 Division of Tumor Biology & Immunology, The Netherlands Cancer Institute, Amsterdam, The Netherlands; 2 Oncode Institute, Utrecht, The Netherlands; 3 Department of Immunology, Leiden University Medical Centre, Leiden, The Netherlands; 4 Division of Molecular Carcinogenesis, The Netherlands Cancer Institute, Amsterdam, The Netherlands; 5 Division of Molecular Pathology, The Netherlands Cancer Institute, Amsterdam, The Netherlands; 6 Genomics Core Facility, The Netherlands Cancer Institute, Amsterdam, The Netherlands; 7 Proteomics Facility, The Netherlands Cancer Institute, Amsterdam, The Netherlands; 8 Department of Medical Oncology, Haaglanden Medical Center, The Hague, The Netherlands; 9 Department of Medical Oncology, The Netherlands Cancer Institute, Amsterdam, The Netherlands; 10 Present Address: Department of Internal Medicine, Groene Hart hospital, Gouda, The Netherlands

**Keywords:** Breast cancer, Tumour immunology

## Abstract

Cancer disrupts intratumoral innate-adaptive immune crosstalk, but how the systemic immune landscape evolves during breast cancer progression remains unclear. We profiled circulating immune cells in stage I–III and stage IV triple-negative breast cancer (TNBC) patients and healthy donors (HDs). Metastatic TNBC (mTNBC) patients had reduced T cells, dendritic cells, and differentiated B cells compared to non-metastatic TNBC patients and HDs, partly linked to prior chemotherapy. Vδ1 γδ T cells from mTNBC patients produced more IL17 than those from HDs. Chemotherapy-naïve mTNBC patients showed increased classical monocytes and neutrophils. Transcriptional, proteomic, and functional analyses revealed that neutrophils in mTNBC exhibited enhanced migratory capacity, elevated granule proteins, and higher ROS production. Some immune changes, such as reduced non-switched B cells and heightened neutrophil migration, were evident in earlier TNBC stages. This study comprehensively maps systemic immunity in TNBC, guiding future research on patient stratification and immunomodulation strategies.

## Introduction

Over the past decade, immunotherapy has revolutionized cancer treatment by targeting the immune system. While much research has focused on local immune responses within the tumor microenvironment (TME), effective antitumor immunity requires ongoing coordination with the peripheral immune system. There is a growing recognition that solid tumors have profound effects on the immune system, significantly altering the overall immune landscape beyond the TME^1–4^. Thus, a comprehensive understanding of cancer immunology must encompass the phenotypic and functional analysis of immune cell lineages in the peripheral immune system. Soluble mediators produced by cancer cells and other cells in the TME can induce the systemic expansion and polarization of myeloid cells, leading to chronic, systemic inflammation^4–10^. Depending on the context, this tumor-induced inflammation can either initiate or support tumor growth^11^ and impact the therapeutic efficacy of systemic treatments^12^.

Tumor-induced systemic inflammation can be characterized by increased neutrophil counts in blood, often represented in the clinic by the neutrophil-to-lymphocyte ratio (NLR). Clinical studies have shown that a high NLR correlates with unfavorable disease outcomes and poor therapy response across many cancer types, including breast cancer^13–18^. Preclinical studies have demonstrated that neutrophils support metastasis formation through diverse mechanisms, including inducing systemic immune suppression, supporting circulating cancer cells, fostering the establishment of the (pre-)metastatic niche, facilitating cancer cell infiltration into distant tissues, and awakening dormant cancer cells^19–27^. In addition to neutrophils, tumor progression has been reported to elicit systemic expansion of monocytes and their reprogramming into an immunosuppressive phenotype^4,28–31^. Furthermore, it has been shown in mouse models and patients with breast and pancreatic cancer that dendritic cell (DC) differentiation is reduced in the bone marrow, leading to a reduction of the systemic cDC1 pool^32^. This could negatively affect antitumor immunity since DCs are the most effective antigen-presenting cells and crucial for T-cell activation. Tumor progression is also often associated with systemic lymphocyte perturbations, characterized by increased regulatory T cell (Treg) frequencies^33–35^, and reduced CD8^+^ and conventional CD4^+^ T cells in the blood of cancer patients^36,37^. Collectively, these data show that cancer influences circulating immune cell populations, which may impact disease progression and (immuno)therapy response. The majority of studies examine relative frequencies and rely on PBMC samples that lack granulocytes and, therefore, do not represent the entirety of the circulating immune system. How absolute cell counts and abundances relative to all immune cells change during disease progression, and how different stages of cancer affect their functionality is largely unclear. Although immune checkpoint blockade (ICB) has created a revolution in oncology, the majority of patients still do not benefit from ICB, including most of the advanced breast cancer patients. A better understanding of the systemic immune landscape is of critical importance to improve immunomodulatory treatments of cancer patients.

**Fig. 1:**
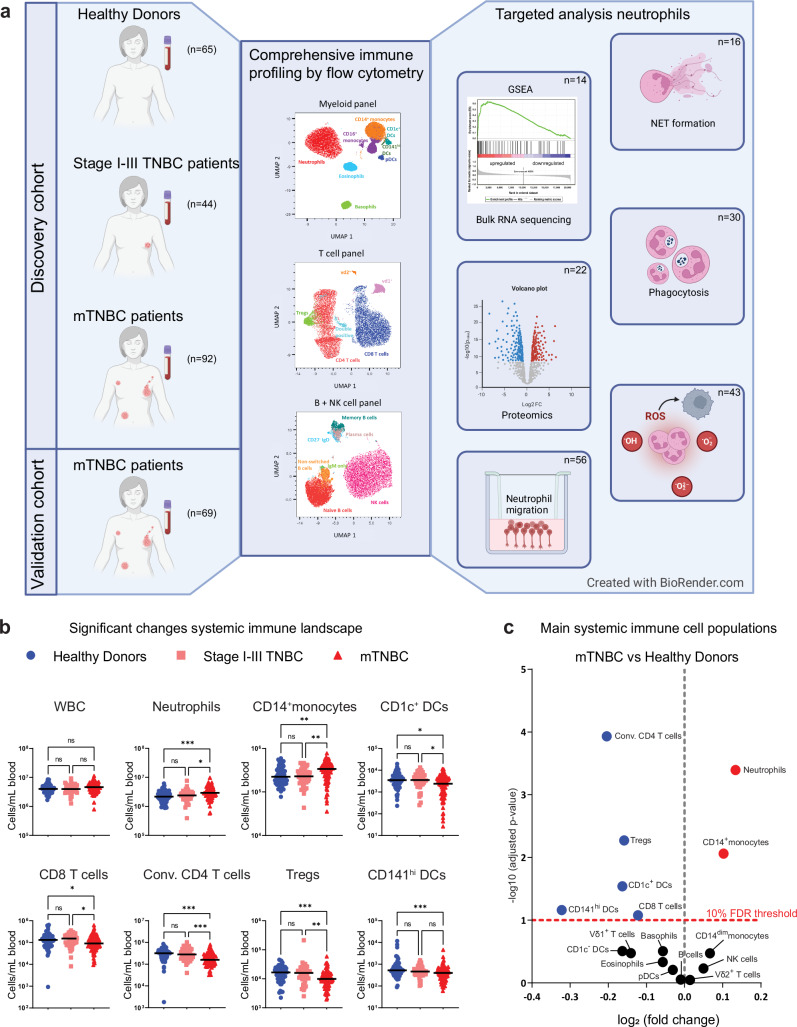
Comprehensive immune profiling of the systemic immune landscape in healthy donors, patients with stage I–III and metastatic triple-negative breast cancer. **a** Graphical summary of included human blood samples and a schematic overview of conducted experiments. **b** Overview of circulating immune cell populations that were significantly dysregulated in patients with triple-negative breast cancer (TNBC). Depicted are cell counts per mL blood as assessed by flow cytometry for healthy donors (HDs; *n* = 65), stage I–III (Stage I–III TNBC; *n* = 44), and metastatic TNBC patients (mTNBC; *n* = 92). The y-axis is on a log scale. *P* values were computed with the Kruskal–Wallis test followed by Dunn’s multiple comparisons correction across groups. **c** Volcano plot summarizing major immune cell populations in blood accessed by flow cytometry comparing HDs to mTNBC patients. The x-axis represents log2 fold changes (red is more abundant in patients with mTNBC, blue is less abundant in patients with mTNBC) and the y-axis represents adjusted *p* values on a negative log10 scale. *P* values are corrected using the Benjamini–Hochberg procedure across immune cell types.

In this study, we set out to extensively profile the systemic immune landscape, including granulocytes, of patients with stage I–III TNBC, patients with mTNBC, and a healthy donor (HD) control group (Fig. 1a). We performed high-dimensional flow cytometry on fresh peripheral blood samples to assess the quantity and quality of circulating immune cell subpopulations. Our data revealed that patients with mTNBC—and to a lesser extent, patients with stage I–III TNBC—have a markedly different systemic immune landscape compared to HDs. We found T cells, DC subsets, and B cell differentiation to be decreased in the blood of patients with mTNBC. In contrast, classical monocytes and neutrophils were increased. A substantial proportion of the included mTNBC patients received prior chemotherapy for their primary tumor, which allowed us to explore the changes in the systemic immune landscape that are associated with chemotherapy. Our findings suggest that the systemic reduction in T cell- and DC subsets in patients with mTNBC could be associated with recent chemotherapy. Conversely, the increase of classical monocytes and neutrophils was purely disease-related. Transcriptomic, proteomic, and functional analysis revealed that neutrophils from patients with TNBC have increased migratory capacity, contain more granule proteins and produce more reactive oxygen species (ROS) than neutrophils from HDs, indicating that neutrophils are not only more abundant in the circulation of mTNBC patients, but also have distinct phenotypic and functional characteristics.

Overall, this study provides the first comprehensive characterization of the systemic immune landscape, including granulocytes, in a large cohort of patients with TNBC compared to HDs. Our data highlight the substantial impact of TNBC and its disease stage on the systemic immune composition and function. This extensive analysis, which includes an independent validation cohort, offers novel insights into the immune profiles specific to patients with TNBC, thereby distinguishing between patients with and without prior chemotherapy treatment. Our data serve as a valuable resource for the field, guiding future preclinical and clinical research and paving the way for immunomodulatory treatment strategies.

## Results

### Metastatic TNBC reshapes the systemic immune landscape

To gain insights into how TNBC influences the systemic immune landscape during disease progression, we performed high-dimensional flow cytometry on fresh peripheral blood samples (Supplementary Fig. 1a–d). We established a dedicated pipeline for the analysis of fresh blood samples^38^, enabling a comprehensive interrogation of the full complexity of the systemic immune landscape, including granulocytes—cell types that are typically lost when working with peripheral blood mononuclear cell (PBMC) samples. We profiled patients with TNBC without distant metastases (stage I–III TNBC (*n* = 44)) and stage IV patients with distant metastases (mTNBC (*n* = 92)). As a control group, we profiled healthy donors (HDs (*n* = 65)) that were age-matched to mTNBC patients and sex- and BMI-matched to all TNBC patients (Supplementary Fig. 2a and Supplementary Table 1). A separate cohort of patients with mTNBC (*n* = 69) was used to validate our main findings (Fig. 1a). Given that leukocyte numbers in the blood are circadian^39,40^, we sought to withdraw blood in the morning. There was no statistically significant difference in time of blood draw between HD and patients with TNBC (Supplementary Fig. 2a).

No differences in total white blood cell (WBC) counts were found between patients with stage I–III TNBC, patients with mTNBC and HDs (Fig. 1b). Most significant differences were observed between HDs and patients with mTNBC, and between patients with non-metastatic versus metastatic disease (Fig. 1b), indicating that the systemic immune system may become more dysregulated as disease progresses. We observed increased neutrophils in patients with mTNBC compared to HDs both in absolute cell counts (Fig. 1b, c) and as relative frequency among WBCs (Supplementary Fig. 3). We additionally observed an elevated NLR in patients with mTNBC compared to patients with stage I–III TNBC and HD (Supplementary Fig. 2c), which is consistent with literature describing elevated NLR in patients with various types of stage IV cancer^18^. Although not statistically significant, we observed a trend towards increased neutrophils in patients with stage I–III TNBC compared to HDs, suggesting that neutrophils increase as the disease progresses (Fig. 1b and Supplementary Fig. 3). Additionally, we found CD14^+^ monocytes to be significantly increased in patients with mTNBC compared to both HDs and patients with stage I–III TNBC (Fig. 1b, c and Supplementary Fig. 3). It was previously reported that breast cancer and pancreatic cancer alter the balance of monocytes and neutrophils compared to antigen-presenting cDC1s in bone marrow and blood^32^. In line with this, we found that the frequencies and cell counts of CD141^hi^ DCs (cDC1s) and CD1c^+^ DCs (cDC2s) were decreased in patients with mTNBC compared to HDs (Fig. 1b, c and Supplementary Fig. 3). cDCs are critical for cytotoxic T-cell activation, and CD141^hi^ DCs are important for cross-presentation^41–43^. Others have shown in preclinical mouse models and patients with pancreatic cancer, that reduced numbers of (pre-)cDCs in blood and the TME are correlated with poor clinical outcome^32,44^. Hence these reduced numbers we found in patients with mTNBC could potentially have negative implications for inducing an adequate immune response.

Within the circulating lymphoid compartment we found that the total counts and frequencies of CD8^+^ T cells, conventional CD4^+^ T cells and Tregs were reduced in patients with mTNBC compared to stage I–III TNBC patients and HDs (Fig. 1b, c and Supplementary Fig. 3). Conversely, γδ-T cell subsets Vδ1 and Vδ2 T cells were unchanged in patients with mTNBC compared to HDs. Interestingly, Vδ2 T cells were increased in stage I–III TNBC patients (Supplementary Fig. 2b) compared to HDs, but this difference was lost in the metastatic setting. No significant differences were found in cell counts and frequencies of CD1c negative DCs, plasmacytoid DCs (pDCs), natural killer (NK) cells, total B cells, and eosinophils between any of the groups (Supplementary Fig. 2). Overall, the frequency plots revealed similar trends (Supplementary Fig. 3). Using an independent validation cohort of patients with mTNBC (*n* = 69) we were able to validate all perturbations to the systemic immune landscape between mTNBC and HDs (Supplementary Fig. 4a). Altogether, our data demonstrate multiple differentially regulated main immune cell populations in patients with mTNBC compared to HDs and patients with stage I–III TNBC.

### IL17 production by Vδ1 γδ T cells is increased in patients with mTNBC

**Fig. 2:**
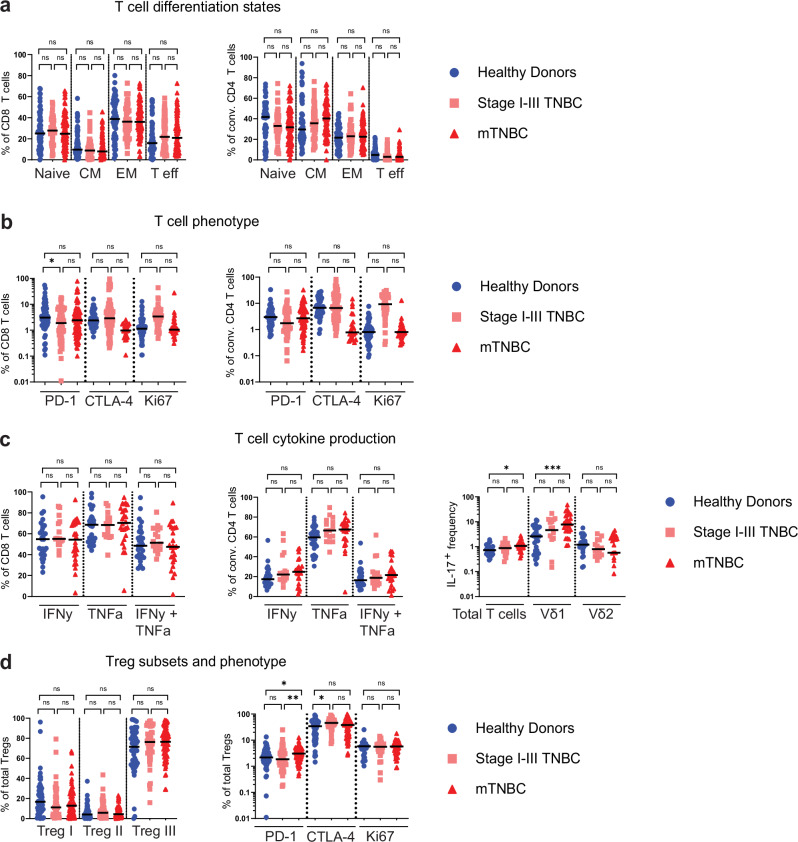
Characterization of circulating T cell subsets. **a** T cell differentiation state based on surface marker expression of CD45RA and CCR7 (see Supplementary Fig. 1c), comparing proportions within conventional CD4+ and CD8 + T cells for HD (*n* = 65), stage I–III TNBC (*n* = 44) and mTNBC (*n* = 92). CM central memory, EM effector memory, and T eff effector T cells. **b** T cell phenotype comparing fractions within CD8+ and conventional CD4 + T cells for HD (*n* = 23), stage I–III TNBC (*n* = 32) and mTNBC (*n* = 52). **c** IFNγ and TNFα production by CD8+ and conventional CD4 + T cells, and IL17 expression on Total T cells and γδ T cells subsets Vδ1 and Vδ2. Determined by flow cytometry for HDs (*n* = 29), stage I–III TNBC patients (*n* = 16), and mTNBC patients (*n* = 26). **d** Regulatory T cell subsets and phenotype comparing HD (*n* = 23), stage I–III TNBC (*n* = 32) and mTNBC (*n* = 52). *P* values for **a**–**d** are computed with the Kruskal–Wallis test followed by Dunn’s multiple comparisons test.

Because total cell counts of CD8^+^, conventional CD4^+^, and regulatory T cells were decreased in patients with mTNBC (Fig. 1), we wanted to investigate the composition and activation status of the circulating T lymphocyte pool in relation to the disease stage. Analysis of the T cell differentiation state (Supplementary Fig. 1c) revealed no differences between patients with stage I–III TNBC, patients with mTNBC and HDs (Fig. 2a). Next, we profiled T cell phenotype and functional state by assessing the expression of the proliferation marker Ki67, PD-1, and CTLA-4, and ex vivo cytokine production of CD8^+^ and CD4^+^ T cells. Across different patients and HDs, there was notable heterogeneity in the peripheral CD8^+^ and conventional CD4^+^ T cell phenotype, which appeared largely unaffected by the presence of TNBC (Fig. 2b). Additionally, the ability to produce IFNγ and TNFα by CD8^+^ and conventional CD4^+^ T cells upon stimulation with PMA-ionomycin for three hours was unaffected by TNBC (Fig. 2c), suggesting that T cells from patients retained similar potential to produce those cytokines ex vivo. Next, we wanted to investigate whether we could retrieve certain aspects of the γδ T cell-IL17-neutrophil axis, that was previously described to drive systemic expansion of neutrophils and metastasis formation in distant organs in mice^19^. Here, we indeed confirm that circulating T cells from patients with mTNBC produced more IL17, with Vδ1-, but not Vδ2, γδ T cells showing a particularly pronounced increase in IL17 production upon ex vivo stimulation (Fig. 2c). Moreover, this intriguing finding could be confirmed in the validation cohort consisting of patients with mTNBC (Supplementary Fig. 4b).

Based on literature^45–47^, we classified circulating Tregs into three subsets based on CD45RA and FoxP3 intensity, with Treg I expressing high levels of CD45RA and intermediate levels of FoxP3 (also referred to as “naïve Tregs”), Treg II expressing low levels of CD45RA and high levels of FoxP3 (also referred to as “activated Tregs”) and Treg III expressing low levels of CD45RA and intermediate levels of FoxP3 (also referred to as “activated non-Treg”) (Supplementary Fig. 1c). We did not observe differences in Treg subset distribution in relation to TNBC status (Fig. 2d). However, patients with mTNBC had a higher frequency of PD-1 positive circulating Tregs relative to HDs and patients with stage I–III TNBC (Fig. 2d). In addition, Tregs in patients with stage I–III TNBC expressed more CTLA-4 compared to HDs (Fig. 2d).

In summary, we revealed equal capability of T cells from patients with (m)TNBC and HDs to produce IFNγ and TNFα when stimulated ex vivo. Notably, we found that IL17 was significantly more produced by Vδ1 γδ T cells from patients with mTNBC.

### Reduced circulating differentiated B cell subsets in patients with mTNBC

**Fig. 3:**
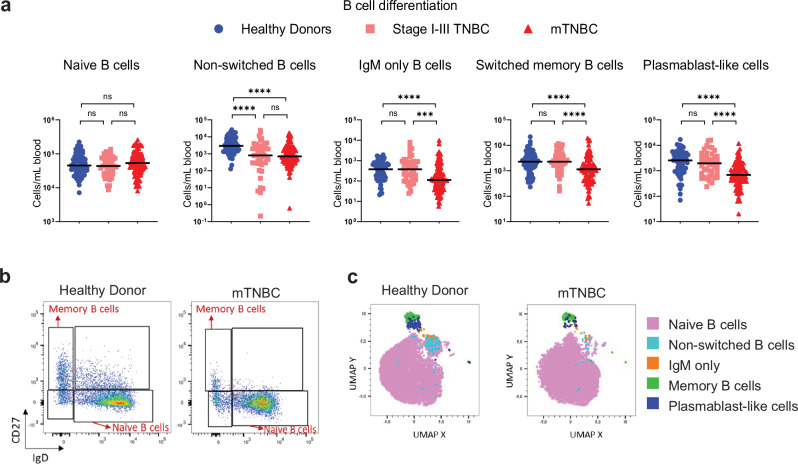
Reduced differentiated B cell subsets in the blood of patients with metastatic triple-negative breast cancer compared to healthy donors. **a** Absolute counts per ml blood for B cell subsets identified using flow cytometry, split according to HD (*n* = 65), stage I–III TNBC (*n* = 44), and mTNBC (*n* = 92). The y-axis is on a log scale. *P* values are computed with the Kruskal–Wallis test followed by Dunn’s multiple comparisons test to obtain adjusted *p* values. **b** Representative dot plots of naïve and differentiated B cells in HD and mTNBC. **c** UMAP plot demonstrating the B cell subset distribution of a representative HD and a patient with mTNBC that were analyzed by flow cytometry on the same day.

The roles of B cells in tumor development remain largely controversial^48–50^. To understand how metastatic disease affects the circulating B cell compartment we investigated B cell subsets in more detail by flow cytometry. Naive B cells, identified by IgD expression and lack of CD27 expression^51^, make up the largest proportion of circulating B cells and their cell counts remained consistent between patients with stage I–III TNBC, mTNBC, and HDs (Fig. 3a). However, when evaluating the subset distribution within the B cell compartment, we found that the proportion of naive B cells compared to differentiated B cell subsets was elevated in patients with metastatic disease (Supplementary Fig. 5a). Furthermore, we observed reduced cell counts and frequencies of non-class switched memory B cells, IgM-only switched memory B cells, switched memory B cells and CD38^+^ plasmablast-like cells in patients with mTNBC compared to HDs (Fig. 3a, b and Supplementary Fig. 5a). Patients with stage I–III TNBC were found to have a similar B cell subset distribution to HDs, except for non-switched B cells, which were reduced in patients with any stage of TNBC compared to HDs (Fig. 3a). Analysis of the B cell compartment in an independent cohort of 69 patients with mTNBC confirmed reduced differentiated B cell subsets in patients with mTNBC compared to HDs (Supplementary Fig. 5b), emphasizing the validity of our findings.

In summary, our investigation revealed a reduced presence of differentiated B cell subsets—both in absolute numbers and as a proportion within the total B cell population—in the blood of individuals with mTNBC compared to HDs. We observed some of these changes already in patients with stage I–III disease, with even stronger effects noted in those with metastatic disease.

### Prior chemotherapy is associated with transient changes in the systemic immune landscape

Beyond tumor characteristics, the immune context of cancer can be significantly influenced by patient characteristics and treatment history, like prior chemotherapy. Chemotherapy not only targets cancer cells but also rapidly dividing normal cells, such as the hematopoietic stem- and progenitor cells in the bone marrow responsible for immune cell production. Understanding the effects of chemotherapy on the immune system is a complex task, as the impact varies significantly depending on the tumor (sub)type^11^ and the type and dosing schedule of chemotherapeutic agents. However, the impact of chemotherapy on the systemic immune landscape after discontinuation of the treatment remains unclear.

**Fig. 4:**
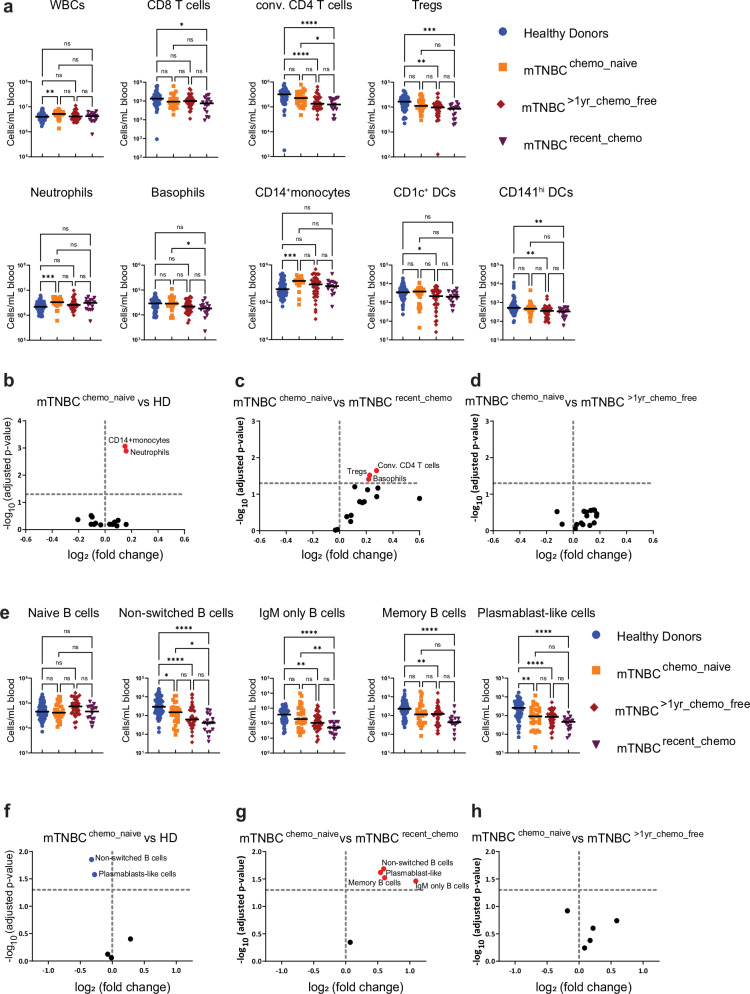
The impact of prior chemotherapy on major immune cell type abundances in the blood of patients with metastatic triple-negative breast cancer. **a** Absolute counts per ml blood of WBC and main circulating immune cells in patients with mTNBC that were split according to treatment history: chemotherapy-naive (mTNBC^chemo_naïve^, *n* = 29), received chemotherapy more than 1 year ago (mTNBC^>1yr_chemo_free^, *n* = 38) or received chemotherapy between 3 and 52 weeks ago mTNBC^recent_chemo^, *n* = 16), and HDs (*n* = 65). The y-axis is on a log scale. (**b**–**d**) Volcano plots summarizing differences in main systemic immune cell counts between **b** HDs and patients with mTNBC that were chemotherapy-naïve, **c** patients with mTNBC that were chemotherapy-naïve and patients with mTNBC that received chemotherapy within the last year, and **d** patients with mTNBC that were chemotherapy-naïve and patients with mTNBC that received chemotherapy more than 1 year ago. **e** Absolute counts per ml blood of differentiated B cell subsets. Patients were split as described in panel (**a**). **f**–**h** Volcano plots summarizing differences in counts of differentiated B cell subset between **f** HDs and patients with mTNBC that were chemotherapy-naïve, **g** patients with mTNBC that were chemotherapy-naïve and patients with mTNBC that received chemotherapy within the last year, and **h** patients with mTNBC that were chemotherapy-naïve and patients with mTNBC that received chemotherapy more than one year ago. For **a**, **e**, *p* values were computed with the Kruskal–Wallis test followed by Dunn’s multiple comparisons correction across groups. For (**b**–**d**) and (**f**–**h**), *p* values are corrected using the Benjamini–Hochberg procedure across immune cell populations.

To explore the impact of chemotherapy that was previously administered to treat the primary tumor (Supplementary Table 2), we stratified mTNBC patients (Figs. 1–3) based on their treatment history. Patients were divided into three groups: chemotherapy-naïve (mTNBC^chemo_naïve^, *n* = 29); last dose of (neo-)adjuvant chemotherapy more than one year ago (mTNBC^>1yr_chemo_free^, *n* = 38), and last dose of (neo-)adjuvant chemotherapy between 3 weeks and 1 year ago (mTNBC^recent_chemo^, *n* = 16). This stratification revealed that mTNBC^chemo_naïve^ patients exhibited elevated total leukocyte counts compared to HDs; a phenomenon that was not observed in patients who had previously received chemotherapy (Fig. 4a), implying that this increase was disease-driven and mitigated by prior chemotherapy. Similarly, we found that neutrophil and CD14^+^ monocyte counts were significantly increased in mTNBC^chemo_naïve^ patients when compared to HDs (Fig. 4a–b), demonstrating that the systemic increase in neutrophil and classical monocyte counts was mTNBC induced, and not chemotherapy-related. In contrast, although our initial analysis revealed reduced CD8^+^ T cells, conventional CD4^+^ T cells, Tregs, CD1c+ DCs, and CD141^hi^ DCs in patients with mTNBC (Fig. 1b), stratification based on prior chemotherapy treatment revealed that these differences were predominantly driven by patients who had previously been treated with chemotherapy (Fig. 4a, b). Prior chemotherapy was not associated with T cell differentiation state, phenotype, and Treg subset distribution (Supplementary Fig. 6b–d). Comparing B cell subsets between mTNBC^chemo_naive^ patients to HDs revealed a significant reduction in non-switched B cells and plasmablast-like cells (Fig. 4e, f), but not IgM only—and memory B cells, suggesting that the reduced differentiated B cell subsets we identified (Fig. 3) were partly cancer-driven and partly impacted by prior chemotherapy.

To study the effects of recent chemotherapy on the systemic immune system, we compared mTNBC^chemo_naïve^ to mTNBC^recent_chemo^. We observed increased cell counts for conventional CD4^+^ T cells and basophils in the mTNBC^chemo_naïve^ group compared to the mTNBC^recent_chemo^ group (Fig. 4a–c), indicating that chemotherapy continues to have a profound effect on the systemic immune landscape after a washout period of at least 3 weeks (to 1 year). Furthermore, we found reduced numbers of IgM only—and switched memory B cells in the mTNBC^recent_chemo^ group, suggesting that previous chemotherapy treatment and TNBC both contributed to the dysregulated B cell pool in the blood of patients with TNBC (Fig. 4e, g). The apparent reduction of Tregs, plasmablast-like cells, and memory B cells in mTNBC^recent_chemo^ compared to mTNBC^chemo_naïve^ (Fig. 4c, g) was not statistically significant when examining individual cell types (Fig. 4a, e) despite matching raw *p* values, due to the necessity of applying different multiple testing corrections.

In order to study the long-term effects of chemotherapy on the systemic immune landscape, we compared mTNBC^chemo_naïve^ to mTNBC^>1yr_chemo_free^ and did not find any of the main immune cell populations to remain perturbed (Fig. 4a, d). Additionally, we demonstrated that B cell differentiation in the mTNBC^>1yr_chemo_free^ group resembled levels found in the mTNBC^chemo_naive^ group (Fig. 4e, h). In conclusion, our data indicate that within this cohort, prior chemotherapy did not have significant long-term effects on the relative and absolute abundances of circulating immune cell populations.

We observed an overall declining trend in cell counts from mTNBC^chemo_naïve^, to mTNBC^>1yr_chemo_free^, to mTNBC^recent_chemo^ for most cell types or subsets (Fig. 4a, e). Consistently, we did not find any of the immune cell populations to be significantly more abundant in patients with mTNBC that received chemotherapy (regardless of the washout period) compared to the mTNBC^chemo_naïve^ group (Fig. 4c, d), aligning with the idea that chemotherapy has an overall depleting effect on proliferating progenitors. Collectively, our data imply that prior chemotherapy influences the composition of the systemic immune landscape by reducing cell counts of basophils, conventional CD4^+^ T cells, IgM-only B cells, and non-switched memory B cells. Importantly, these changes are not detectable in the group that received the last dose of chemotherapy more than 1 year ago, suggesting that chemotherapy-induced changes are not persistent in patients with mTNBC. In contrast, systemic increases in neutrophils and monocytes are disease-driven.

### Neutrophils from patients with TNBC have enhanced migratory capacity

**Fig. 5:**
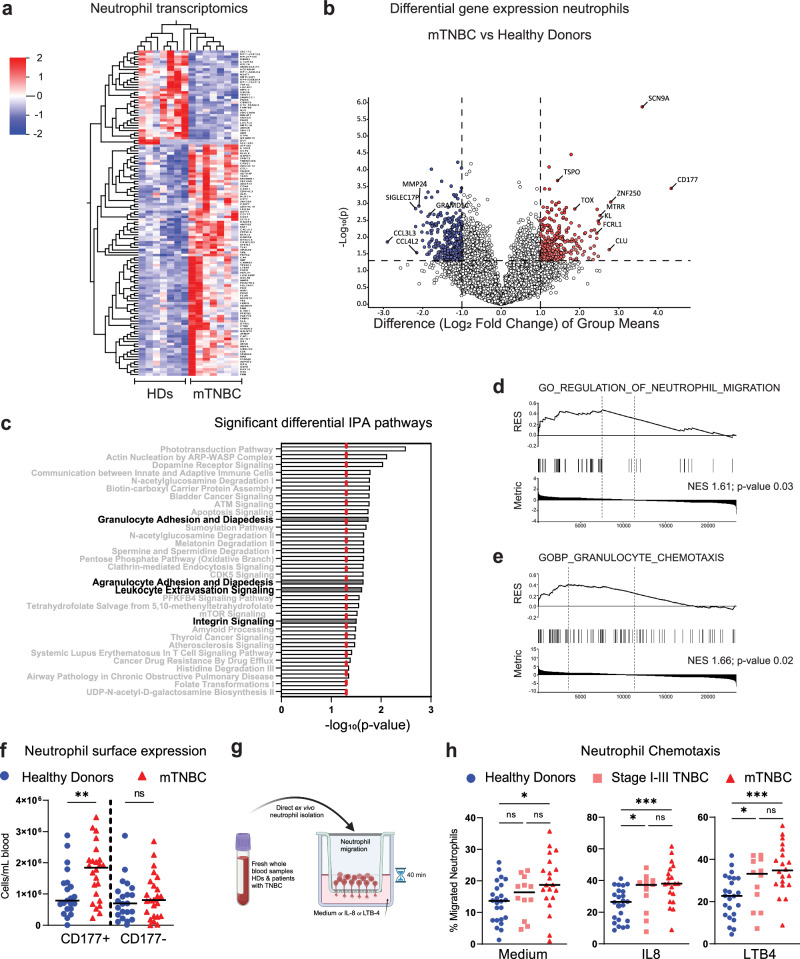
Neutrophils derived from individuals with triple-negative breast cancer exhibit heightened migratory capabilities. **a** Bulk RNA-sequencing data of purified blood neutrophils from patients with HDs (*n* = 7) and mTNBC (*n* = 7). Heatmap visualizing the differentially expressed genes between HDs and patients mTNBC. Colors indicate the row Z-score ranging from 2 to −2. **b** Volcano plot showing fold changes for genes described in (**a**). **c** All statistically significant pathways that came out of the ingenuity pathway analysis on differentially expressed genes between neutrophils from HDs and patients with mTNBC. **d**, **e** Gene set enrichment analysis performed on the same neutrophil bulk RNA-sequencing dataset, with **d** GO gene set “regulation of neutrophil migration” and **e** GO gene set “granulocyte chemotaxis”. **f** Surface marker expression of CD177 determined by flow cytometry on neutrophils from HDs (*n* = 21) and patients with mTNBC (*n* = 25). *P* values are computed with an ANOVA followed by Šídák’s multiple comparisons test. **g**, **h** Neutrophil migration rates determined in direct ex vivo chemotaxis assays using IL-8 and LTB-4 as chemo-attractants. Neutrophils were processed immediately after blood draws from HDs (*n* = 24), stage I–III TNBC patients (*n* = 12) and mTNBC patients (*n* = 20). *P* values are computed with the Mann–Whitney *U*-test.

Our comprehensive immune profiling analysis demonstrated that neutrophils are the most increased cell population in the circulation of chemotherapy-naive patients with mTNBC (Fig. 4a, b). Previous research demonstrated that tumor-induced neutrophils promote mammary tumor progression and metastatic spread in preclinical mouse models^10,19,25,52,53^ and that TNBC patients with increased NLR have a worse clinical prognosis^54^. We, therefore, hypothesized that neutrophils in the blood of patients with mTNBC are phenotypically and functionally different from neutrophils in HDs. To test this hypothesis, we interrogated the transcriptome profile of freshly isolated circulating neutrophils from seven patients with mTNBC and seven HDs by bulk RNA-sequencing. We identified 90 upregulated and 37 downregulated genes between neutrophils from patients with mTNBC and HDs (adjusted *p* value <0.05) (Fig. 5a, b). In silico pathway analysis indicated that, among the various differential pathways, multiple pathways related to neutrophil migration were significantly enriched in neutrophils from patients with mTNBC compared to those from HDs (Fig. 5c). Gene set enrichment analysis (GSEA) similarly indicated that neutrophils from patients with mTNBC were enriched for genes involved in migration (Fig. 5d, e). One of the most upregulated genes was *CD177* (Fig. 5b), encoding a glycosyl-phosphatidylinositol (GPI)-linked cell surface glycoprotein. CD177 is expressed by activated neutrophils, is upregulated in inflammatory settings, and modulates neutrophil migration^55,56^. Since *CD177* is an important driver of the migration signature, we sought to confirm whether the increased *CD177* transcription in neutrophils from patients with mTNBC corresponds to differences in protein levels at the cell surface. Using flow cytometry analysis, we verified in an independent set of patients with mTNBC a significantly higher number of CD177 positive neutrophils compared to HDs; this difference was not observed for CD177 negative neutrophils (Fig. 5f).

To functionally validate the predicted enhanced migratory capacity of neutrophils from patients with mTNBC, we performed transwell migration assays. The results confirmed the increased migratory capacity of circulating neutrophils from an independent set of mTNBC patients compared to those from HDs, even in the absence of chemo-attractants. This effect was further heightened in the presence of chemo-attractants (Fig. 5g, h). Importantly, neutrophils from stage I–III TNBC patients already exhibited increased migration towards chemo-attractants compared to neutrophils from HDs, indicating that this altered neutrophil behavior is instigated during early or locally advanced disease stage and maintained during disease progression. To conclude, our findings demonstrate that circulating neutrophils from patients with TNBC have greater migratory capacity compared to neutrophils from HDs.

### Neutrophils from patients with mTNBC contain more granule proteins and produce more ROS

**Fig. 6:**
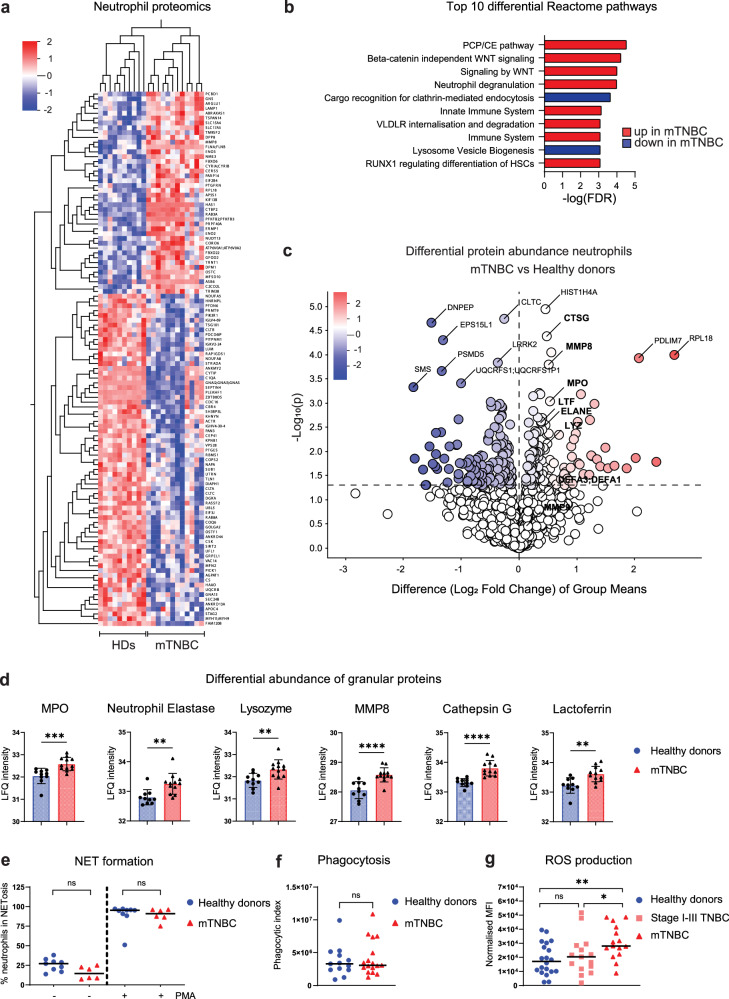
Altered proteome and more ROS production by circulating neutrophils from patients with metastatic triple-negative breast cancer compared to healthy donors. **a** Heatmap visualizing differentially abundant proteins between freshly isolated blood neutrophils from HDs (*n* = 10) and mTNBC patients (*n* = 12). Colors indicate the row Z-score ranging from 2 to −2. **b** Top ten Reactome pathways representing the functional domains of the differentially abundant proteins between neutrophils from HDs and patients with mTNBC. **c** Volcano plot showing log fold changes of differentially abundant proteins (*p* < 0,05) and highlighting all granule proteins of the dataset in bold. Additionally, proteins with the highest fold change and lowest *p* value are labeled (not in bold). **d** Quantification of the significantly differentially abundant granule proteins from the proteomics dataset between HDs and patients with mTNBC. *P* values are computed with the Mann–Whitney *U*-test. **e** Proportion of blood neutrophils from patients with mTNBC and HDs undergoing ex vivo NETosis with and without PMA stimulation. **f** Phagocytic index representing an opsonizing rate of *E.coli* BioParticles by freshly isolated blood neutrophils from patients with mTNBC and HDs. **g** Reactive oxygen species (ROS) production by neutrophils isolated from fresh blood samples of HDs (*n* = 20), patients with stage I–III TNBC (*n* = 15) and patients with mTNBC (*n* = 15). *P* values are computed with the Mann–Whitney *U*-test.

To further investigate the effects of mTNBC on the functional state of circulating neutrophils, we performed a full proteomic analysis comparing neutrophils from patients with mTNBC and neutrophils from HDs. We identified a total of 111 differentially regulated proteins (adjusted *p* < 0.05): 42 upregulated and 69 downregulated proteins in neutrophils from patients with mTNBC compared to HDs (Fig. 6a). Reactome analysis identified various up- and down-regulated pathways, including a significant increase in proteins involved in neutrophil degranulation in neutrophils from patients with mTNBC (Fig. 6b). This pathway consists of 450 proteins that are important for neutrophil vesicle exocytosis, as well as proteins present in those vesicles. In addition, we found that neutrophil granule proteins were significantly enriched in neutrophils from patients with mTNBC (Fisher’s exact test, *p* = 0.021) (Fig. 6c, d).

We next assessed several additional important effector functions of neutrophils, and how they are influenced by TNBC, including neutrophil extracellular trap (NETs) formation, phagocytosis, and ROS production. We found no differences in ex vivo NET-formation, either spontaneously or after PMA stimulation (Fig. 6e) or phagocytic ability in neutrophils from patients with mTNBC compared to HDs (Fig. 6f). In contrast, we found that neutrophils from patients with mTNBC produce significantly more ROS than HD neutrophils (Fig. 6g). Furthermore, there was a modest trend toward increased ROS production by neutrophils from stage I–III TNBC patients compared with HDs, which could indicate a gradual change in the functional phenotype of neutrophils as the disease progresses. According to existing data^57^, increased levels of ROS from neutrophils could potentially exert an immunosuppressive influence. Although the patient numbers were insufficient to statistically test the effect of prior chemotherapy treatment on ex vivo migration- and ROS production capacity of neutrophils, we observed no clear separation of the patients based on chemotherapy treatment history (Supplementary Fig. 7a, b).

In summary, these results show that neutrophils from patients with mTNBC contain more granule proteins and produce more ROS compared to neutrophils from HDs, indicating that patient neutrophils are not only more abundant and transcriptionally distinct but are also functionally altered by TNBC.

## Discussion

The impact of solid tumors on the overall systemic immune landscape during cancer progression is not fully understood. This study aimed to investigate the changes in the systemic immune landscape at different TNBC stages. Additionally, we explored how prior chemotherapy treatment could be associated with changes in the systemic immune landscape in the metastatic setting. We applied multi-parameter flow cytometry to comprehensively assess the abundance, phenotype, and activation states of both lymphoid and myeloid immune populations from fresh peripheral blood samples. Preclinical evidence strongly implicates a critical role for neutrophils in disease progression^13–16,58–62^. However, these fragile cells are often overlooked due to the fact they cannot be stored. By analyzing fresh blood samples, we were able to capture the full complexity of the immune landscape, including all granulocytes, and we were able to perform in-depth functional neutrophil analyses.

Our data established that the disease stage had a major impact on the systemic immune composition and function in patients with TNBC. We demonstrated that patients with mTNBC manifested lower levels of circulating T cells, DC subsets, and differentiated B cells. In contrast, classical monocyte and neutrophil counts and frequencies were higher in mTNBC patients compared to HDs. When subjecting circulating neutrophils to more qualitative analysis, we revealed that neutrophils from patients with mTNBC had heightened transcription of genes associated with neutrophil trafficking, showed an increased ex vivo migratory capacity, presented elevated levels of granule proteins, and had increased ROS production. While no apparent changes in cell counts or frequencies were observed in patients with stage I–III TNBC, alterations in neutrophil functionality, and in particular in migratory capacity, did emerge in the non-metastatic disease setting.

In more detail, within the circulating lymphocyte compartment we observed that Tregs in patients with stage I–III TNBC expressed more CTLA-4 compared to HDs, something that was not observed in patients with mTNBC. This raises the question of whether Tregs in stage I–III TNBC patients have a more immunosuppressive phenotype than at the metastatic stage, and may contribute to systemic immunosuppression. Additionally, we observed a reduction in CD8^+^ T cells, conventional CD4^+^ T cells, and Tregs in patients with mTNBC compared to HDs, which seemed predominantly associated with prior chemotherapy. Others have previously discussed lymphocyte repopulation dynamics after chemotherapy, noting that NK T cells and CD8^+^ T cells return to pre-chemotherapy levels within a year. However, B cells remain significantly lower after 9 months, and memory CD4^+^ T cells exhibit an abnormal bias toward inflammatory effectors that persists for years, albeit in cohorts of breast cancer patients with mixed or unknown molecular subtypes^63,64^. To the best of our knowledge, we are the first to explore the effects of chemotherapy on the systemic immune landscape in its full complexity more than one year after the last chemotherapy administration. Notably, we observed no differences in CD8^+^ T cell and Treg counts between chemotherapy-naïve mTNBC patients and those who received chemotherapy recently. However, the combined effect of having mTNBC and recent chemotherapy significantly reduced CD8^+^ T cell and Treg counts compared to HDs. These cumulative impacts on the overall immune status of patients may carry substantial clinical consequences, including diminished vaccine responses, and heightened infection risks^65^, and are likely to influence the efficacy of cancer immunotherapy, given the pivotal role of CD8^+^ T cells in anticancer immunity^66^. When investigating the functional consequences of TNBC on T cells, we uncovered that Vδ1 γδ-T cells from patients with mTNBC produced more IL17 compared to those from HDs, which seemed independent from chemotherapy treatment history, along with increased circulating neutrophils. These findings are in line with our published preclinical work showing that IL17-producing γδ-T cells are increased in mammary tumor-bearing mice, and that they drive systemic expansion and polarization of neutrophils towards a CD8^+^ T cell-suppressive phenotype, promoting metastatic spread^19^. Additionally, it has previously been described that HER2- ER+/− breast cancer patients had an increased frequency of Th2/ Th17 cells, based on the surface marker expression of CXCR3, CCR4, and CCR6^67^. We suggest that exploring the clinical application of targeting the IL17 pathway may be of interest. Furthermore, we found that patients with stage I–III TNBC had a lower frequency of PD-1 positive circulating CD8^+^ T cells compared to HDs, while PD-1 expression on Tregs is increased in patients with mTNBC compared to patients with stage I–III TNBC and HDs. These findings suggest differential regulation of PD-1 expression across immune cell subsets in TNBC. Correlation analysis showed no association between tumor TIL scores and the systemic immune profiles of patients with either non-metastatic or metastatic TNBC (data not shown).

Furthermore, patients with mTNBC were found to have an increased frequency of naïve B cells compared to HDs, accompanied by a reduction in differentiated B cell subsets such as memory B cells and plasmablast-like cells. It has previously been reported that elevated frequency of circulating plasmablasts in patients of various cancer types (melanoma, lung, and renal) correlates with improved patient outcomes^68,69^. Moreover, high baseline IgG titers in the blood of melanoma patients showed a positive correlation with response to immune checkpoint blockade^70,71^. Our findings of reduced numbers of differentiated B cells suggest a previously unappreciated impact of chemotherapy and disease stage on the circulating B cell compartment that may have consequences for patients’ humoral immune responses. Further research is needed to understand whether differentiated B cell subsets are decreased in tumors as the disease progresses, since tumor-infiltrated B cells can also have a profound influence on the clinical outcome of TNBC^72^.

Within the myeloid compartment, we observed increased levels of classical CD14^+^ monocytes in patients with mTNBC compared to HDs, reinforcing the notion that cancer induces systemic inflammation. Others previously described that human breast cancer changes classical/non-classical monocyte ratios and alters transcriptional profiles of monocytes^28,73^. Furthermore, a high lymphocyte-to-monocyte ratio and high monocyte frequencies in peripheral blood correlate with poor clinical outcomes in cancer patients^74–77^. Our findings underscore that the dysregulation of classical monocytes represents a progressive disruption of the immune system closely associated with disease progression in TNBC. Importantly, this monocyte dysregulation is a tumor-driven phenomenon. Interestingly, non-classical monocytes, previously associated with enhanced control of metastasizing cells in murine models^78–80^, remain unaffected by these systemic changes.

In our study, we observed a statistically significant increase in circulating neutrophil levels among chemotherapy-naïve patients with mTNBC compared to HDs, which constituted the most pronounced difference in quantity between the two groups. Combining complementary technologies including RNA-sequencing, proteomics, and functional assays, revealed that neutrophils from patients with mTNBC show enhanced migratory capacity. This is in line with published work describing increased neutrophil migration in patients with other cancer types (non-small cell lung cancer and head-and-neck cancer), albeit in small cohorts with mixed disease stages^81,82^. In this study, we show that neutrophils have increased migratory capacity in TNBC in a disease stage-dependent manner. Cell migration is an important feature of neutrophil biology and—in the context of cancer—is a critical component of their ability to prepare the (pre-)metastatic niche and contribute to disease progression^52,83–86^.

Moreover, we found that mTNBC neutrophils produce significantly more ROS than HD neutrophils. ROS produced by neutrophils can exert immunosuppressive effects on T and NK cells, induce DNA damage, and enhance tumor metastasis by disrupting endothelial cell junctions and facilitating extravasation. However, in specific tumor contexts, neutrophils may counteract invasion, partly by inducing cancer cell death through elevated ROS levels^57,87^. Given the context-dependent nature of the effects of ROS, further research is needed on the implications of increased ROS production in patients with TNBC. There is a critical need to normalize the systemic effects of cancer on the immune system, and our data provide valuable insights into the functional changes that are induced by TNBC, which might lay a foundation for future (pre-)clinical studies. For instance, the altered biology of neutrophils suggests that their migration could represent a novel angle for future therapeutic strategies.

Additionally, proteomic analysis identified alterations in neutrophil degranulation pathways and revealed increased abundances of granule proteins like MPO, neutrophil elastase, and lysozyme in neutrophils from patients with mTNBC compared to HDs. Secretion of granules filled with toxic proteins is a key pillar in neutrophils’ effector function and their ability to control invading pathogens. Granules are divided into four subgroups depending on their protein content and synthesis during granulopoiesis^88,89^. Our data did not reveal a specific pattern in the type of granule proteins. Specifically, no cancer-associated enrichment was observed for primary/azurophil, secondary/specific, or tertiary/gelatinase granule proteins. Although the vast majority of (pre-)clinical studies found that tumor-associated neutrophils correlate with poor clinical outcome, neutrophils have also been described to play an anti-tumorigenic role in the TME by direct killing of tumor cells or by interacting with other immune cells^86,89–92^. Our data hint at the preservation of cytotoxic potential in neutrophils from patients with mTNBC, perhaps suggesting that they still have the potency to be mobilized against the tumor. Further investigations are warranted to substantiate the implications of the increase in granule proteins for patients. Since our study reveals increased levels of systemic neutrophils in the metastatic TNBC setting and a progressive alteration of several functional aspects of neutrophils such as increased ROS production and enhanced migration capacity with disease advancement, it is tempting to speculate that these progressive changes contribute to the accumulating clinical data showing immunotherapy exhibits greater efficacy in non-metastatic (breast) cancer compared to late stage disease^93,94^. Encouragingly, FDA (but not EMA) approval for immunotherapy as (neo-)adjuvant therapy has been extended not only to highly immunogenic cancers such as melanoma and non-small cell lung cancer but also to TNBC, offering new avenues for improved treatment strategies.

While elevated NLR is associated with disease progression in various cancers^13–18^, and we observe a significant increase in systemic neutrophils in patients with mTNBC compared to HDs (Fig. 1b, c), it exhibits significant variability, even among healthy individuals, making blood neutrophil abundance an unreliable standalone biomarker for early metastasis or recurrence. However, based on our findings, it is intriguing to speculate that the neutrophil transcriptome or proteome may harbor prognostic signatures with potentially greater specificity and reliability. Further validation studies in larger patient cohorts are needed to explore this hypothesis.

In our cohort, patients with mTNBC who had undergone chemotherapy had received varying types and numbers of chemotherapy lines; a limitation of our study is the insufficient statistical power to analyze patients’ pre-treatments based on specific chemotherapy types or the number of treatment lines administered. Additionally, since this is a retrospective analysis, and the patient cohort was originally not designed to study the impact of chemotherapy on the immune system, we cannot formally rule out a confounding factor arising from potential variations in tumor or patient characteristics between the chemotherapy-naïve and chemotherapy-exposed groups. Nonetheless, given the substantial size of our cohort, and access to historic treatment information, it offers us a unique opportunity to explore the association between prior chemotherapy and the systemic immune landscape. Strengths of our study include the comprehensive approach we took using fresh blood samples, the validation cohort further substantiating our findings, and the assessment of neutrophil functionality in addition to quantitative approaches.

Our data revealed that TNBC profoundly impacts the systemic immune landscape. Furthermore, our data indicate that prior chemotherapy treatment could be associated with systemic immune alterations. When patients with mTNBC had not received chemotherapy for over a year, the levels of immune cells in their blood resemble those of patients with mTNBC who had never undergone chemotherapy. Investigating prospective longitudinal chemotherapy effects on TCR/BCR-repertoire, assessing the functionality of other immune cell types besides neutrophils, and exploring potential epigenetic rewiring are important to fully understand the impact of standard-of-care chemotherapy in breast cancer patients. In the future, dissecting the role of different types of chemotherapy may shed new light on which types and combinations of chemotherapeutic drugs are less impactful for the effector immune system, resulting in a more favorable immune profile.

## Methods

### Patients and healthy donors

TNBC patient blood samples were obtained from patients enrolled in either a clinical trial or biobank protocol, after approval by the local medical ethical committee and/or institutional review board of the Netherlands Cancer Institute. All patients provided informed consent for the current study. Fourteen patients were enrolled in a biobanking protocol of the Netherlands Cancer Institute (CFMPB450); 31 patients were included in the BELLINI trial (stage I–III TNBC, NCT03815890); 91 patients were included in the Triple B trial^95^ (discovery cohort mTNBC, all before first line of palliative treatment, NCT01898117); 69 patients were included in the TONIC trial^38,96^ (validation cohort mTNBC, with no to max three lines of prior treatment, NCT02499367). For samples obtained in the context of a clinical trial, only baseline blood samples were included in the analysis for this paper and the current analyses were not part of the main study plan of the clinical trial. Stage I–III TNBC patients did not receive chemotherapy in the past. From the 92 mTNBC patients in our discovery cohort, 29 patients (32%) did not receive prior chemotherapy treatment, for nine patients it was unknown or the date of the last chemotherapy administration was unknown, and 54 patients (59%) received prior chemotherapy for their primary tumor. Of the pre-treated patients, 38 patients received their last dose of chemotherapy more than 1 year ago, with a median washout period of 2.3 years (range 395–4423 days), and 16 patients received their last dose of chemotherapy less than 1 year ago, with a median washout period of 223 days (range 21–365 days). Both chemotherapy-experienced and chemotherapy-naïve patients had a full range of tumor sizes from T1 to T4 at the time of diagnosis, although a part of the chemotherapy-naïve patients (76%) presented with metastatic disease at the time of diagnosis, which was not the case for chemotherapy-experienced patients. Of note, NK cell markers were added later to the panels, so n-numbers for NK cell analysis are as follows: HD *n* = 23, stage I–III *n* = 29, and mTNBC *n* = 25. All study protocols were conducted in accordance with the ICH Harmonized Tripartite Guideline for Good Clinical Practice and the principles of the Declaration of Helsinki. Fresh blood samples from 53 healthy women (healthy donors, HD) were obtained after approval by the local medical ethical committee (NCT03819829). Additionally, fresh blood samples from 12 healthy women were obtained anonymously from the Dutch national blood transfusion service (Sanquin Blood Supply, Amsterdam, The Netherlands). All patients and healthy donors provided written informed consent before enrollment. Basic clinical characteristics of these cohorts are described in Supplementary Table 1. HDs were age-matched to mTNBC patients (Supplementary Fig. 2a). Blood samples were drawn primarily in the morning (88% was taken before noon) and blood draw times were comparable for HDs, stage I–III TNBC patients and mTNBC patients (Supplementary Fig. 2a).

### Flow cytometry

Blood samples were processed and analyzed within 24 h after blood draw. All samples were processed in the same way, by the same team, and in the same lab. Peripheral blood was collected in EDTA vacutainers (BD) and subjected to red blood cell lysis (lysis buffer: dH2O, NH4Cl, NaHCCO3, EDTA). Cells were resuspended in PBS containing 0.5% BSA and 2 mM EDTA and counted using the NucleoCounter NC-200 (Chemometec) automated cell counter. To obtain absolute leukocyte counts per mL of human blood, the total amount of post-lysis cells was divided by the volume (mL) of blood obtained from the patient (~10 mL). For surface antigen staining, cells were first incubated with human FcR Blocking Reagent (1:100 Miltenyi) for 15 min at 4 °C and then incubated with fluorochrome-conjugated antibodies for 30 min at 4 °C, in the dark. For intracellular antigen staining, cells were fixed with Fixation/Permeabilization solution 1X (Foxp3/Transcription Factor Staining Buffer Set, eBioscience) for 30 min at 4 °C and stained with fluorochrome-conjugated antibodies in Permeabilization buffer 1X (eBioscience) for 30 min at room temperature. Viability was assessed by staining with either 7AAD staining solution (1:10; eBioscience), Zombie Red Fixable Viability Kit (1:800, BioLegend), or Propidium Iodide (Thermo Fisher Scientific). For the analysis of cytokine production, cells were stimulated with PMA (0.25 ng/mL) and Ionomycin (1 nM) in the presence of GolgiPlug for 3 h at 37 °C, 5% CO_2_. After stimulation, cells were prepared according to the intracellular staining protocol described above. Data acquisition was performed on an LSRII SORP flow cytometer (BD Biosciences) using Diva software. To standardize the performance of this machine over time as good as possible, CS&T beads (BD) were used to optimize general performance, and Sphero 8 peaks Rainbow Calibration particles (BD) were used to adjust PMT voltages if necessary. Additionally, single stained compensation controls were taken along for each experiment. Data analysis was performed using FlowJo software version 10.6.2. Flow cytometry antibodies can be found in Supplementary Table 3. Gating strategies are displayed in Supplementary Figs. 1a (Myeloid panel gating), 1b (B and NK cell panel gating), and 1c, d (T cell panel gating). The neutrophils to lymphocyte ratio (NLR) was calculated by dividing neutrophil counts by lymphocyte counts.

### Neutrophil Isolation from fresh blood samples

For bulk RNA-sequencing, neutrophils were FACS isolated on a FACSAria Fusion sorter (BD Biosciences) from fresh peripheral blood samples from seven patients with metastatic TNBC and 7 age- and BMI-matched HDs (see Flow Cytometry paragraph above for staining procedures). Cells were sorted directly into RLT buffer (Qiagen) supplemented with 1% beta-mercaptoethanol and snap-frozen using dry ice and ethanol. For functional assays, neutrophils were isolated from fresh whole blood samples using the human MACSxpress Whole Blood Neutrophil Isolation Kit (Miltenyi Biotec B.V.). Residual red blood cells were lysed using red blood cell lysis buffer (dH2O, NH4Cl, NaHCCO3, EDTA), resulting in a neutrophil suspension with typically >98% purity.

### Bulk RNA-sequencing

We included seven patients with mTNBC (of which 1 (14%) was chemotherapy-naïve, 1 (14%) was chemo-free for more than 1 year, and 5 (72%) received recent chemotherapy) and 7 HDs, without any pre-selection. RNA was isolated from sorted neutrophil samples using the RNeasy Micro Kit (Qiagen), including an on-column DNase digestion (Qiagen), according to the manufacturer’s instructions. Quality and quantity of the total RNA was assessed on the 2100 Bioanalyzer instrument following the manufacturer’s instructions “Agilent RNA 6000 Pico” (Agilent Technologies). In general RNA yields of 5–20 ng total RNA and RNA integrity numbers (RIN) above 8 were obtained. RNA library preparation was performed according to a published protocol by Picelli et al. ^97^ with modifications. In short, 2–7 ng of total RNA for each sample was prepared in a volume of 4 ul. Oligo dT primer hybridization was performed by the addition of Oligo dT mix (0.7 ul H_2_O, 0.1 ul RNAse inhibitor (40 U/ul), 0.1 ul dNTP mix (100 mM), and 0.1 ul Oligo-dT30VN primer (100 uM)). Reverse transcription was performed as described, but the MgCl2 concentration was adjusted to 10 mM. Template switching and 10 (1–4 ng RNA input) or 11 (5–8 ng RNA input) cycles pre-amplification of full-length cDNAs with template switching oligo’s was performed using ISPCR primer at a final concentration of 0.08 uM. The amplified full-length cDNA was used for NGS library construction by Tagmentation for Illumina sequencing, using the Illumina Nextera XT DNA sample preparation kit (Illumina). RNA-sequencing libraries were quantified and normalized based on library QC data generated on the Bioanalyzer system according to the manufacturer’s protocols (Agilent Technologies). A multiplex sequencing pool of all uniquely indexed RNA libraries was composed by equimolar pooling before sequencing on the HiSeq 2500 Illumina sequencing platform. HiSeq 2500 single-end sequencing was performed using 65 cycles for Read 1, 8 cycles for Read i7, using HiSeq SR Cluster Kit v4 cBot (GD-401-4001, Illumina) and HiSeq SBS Kit V4 50 cycle kit (FC-401-4002, Illumina). Almost 95% of the sequenced reads passed the filter, and ~93% of the reads have quality values above Q30. This resulted in, on average, 16 M passing filter reads per sample. All reads passing filters have been used for further analysis. Reads were aligned with Hisat (version 2.1.0), allowing for exon-exon junctions, against the ensembl human build 38. After mapping, on average, 95% of the reads have been mapped to the reference genome. Read counts were generated using Itreecount (https://github.com/NKI-GCF/itreecount), a perl script which gives similar output compared to the HTSeq-count python package. As a reference, ensembl gtf version 87 was used to count the reads. All samples were merged into one dataset. Genes that have zero expression across all samples were removed from the dataset. Data analysis was performed using the DESeq2 package in RStudio under R version 4.1.0 for differential gene expression analysis and Qlucore software (Qlucore Omics Explorer 3.8, Lund, Sweden) for GSEA and visualization purposes. GSEA was performed using the “GOBP_GRANULOCYTE_CHEMOTAXIS” geneset and the “GO_REGULATION_OF_NEUTROPHIL_MIGRATION”^98^.

### Proteomics

Isolated neutrophils from fresh blood samples were washed 3x with PBS, and 1 × 10^6^ cells were frozen and stored at −80 °C until the dataset was complete. We included 12 patients with mTNBC (of which 5 (42%) were chemotherapy-naïve, 3 (25%) were chemo-free for more than 1 year, and 4 (33%) received recent chemotherapy) and 10 HDs, without applying any pre-selection criteria. The proteomic samples were independent from the RNA-sequencing samples. Frozen neutrophil cell pellets from HDs and patients with mTNBC were heated for 10 min. at 95 °C in 1x S-Trap lysis buffer (5% SDS in 50 mM TEAB pH 8.5), followed by sonication. Lysate protein concentrations were determined with a BCA Protein Assay Kit (Thermo Fisher Scientific), proteins were reduced with DTT and alkylated with iodoacetamide, and 50 µg protein amounts were digested o/n with trypsin (Sigma-Aldrich; enzyme/substrate ratio 1:10) on S-Trap Micro spin columns according to the manufacturer’s instructions (ProtiFi, NY, USA). Peptides were eluted, vacuum dried, and stored at −80 °C until LC-MS/MS analysis. LC-MS/MS was performed by nanoLC-MS/MS on an Orbitrap Exploris 480 mass spectrometer (Thermo Fischer Scientific) connected to a Proxeon nLC1200 system. Peptides were directly loaded onto the analytical column (ReproSil-Pur 120 C18-AQ, 2.4 μm, 75 μm × 500 mm, packed in-house) and eluted in a 210-min gradient containing a linear increase from 6 to 23% solvent B (solvent A was 0.1% formic acid/water and solvent B was 0.1% formic acid/80% acetonitrile). The Exploris 480 was run in data-dependent acquisition (DDA) mode, with 2 s. cycle time. Survey scans of peptide precursors from m/z = 375–1500 were acquired in the Orbitrap at 60 K resolution with AGC target and maximum injection time mode set to “Standard” and “Auto”, respectively. Tandem MS was performed by quadrupole isolation at 1.2 Th. followed by HCD fragmentation with normalized collision energy of 30 and Orbitrap MS2 fragment detection at 15 K resolution, AGC target, and maximum injection time mode set the same as described for MS1. Only precursors with charge states 2–6 were sampled for MS2. Monoisotopic precursor selection was turned on; the dynamic exclusion duration was set to 32.5 s with a 10 ppm tolerance around the selected precursor.

Neutrophil proteome data were analyzed with label-free quantification using MaxQuant (version 2.0.1.0)^99,100^ using standard settings. Fragment spectra were searched against the Swissprot human database (version 2021_04; 20,395 entries). Trypsin/P was specified as protease specificity, allowing a maximum of two miscleavages; oxidation (M) and acetyl (protein N-terminus) were selected as variable modifications and carbamidomethylation (C) was selected as fixed modification; for identification, “match between runs” was applied. Protein group abundances were extracted from the MaxQuant proteinGroups.txt file, imported into Perseus (1.6.15.0)^101^, and Log2-transformed. Values were filtered for presence in at least 50% of all samples in either the donor or patient group. Missing values were replaced by an imputation-based normal distribution using a width of 0.3 and a downshift of 1.8. Proteins with *T*-test *p* < 0.05 were considered differential; further data analysis and interpretation was performed using Qlucore software and the Reactome pathway database^102,103^.

### Chemotaxis assay

Purified neutrophils (as described in “Neutrophil Isolation from fresh blood samples”) were stained for 30 min at 37 °C with the cell-permeant dye Calcein acetoxymethyl (Thermo Fisher Scientific) at a final concentration of 1 µM. After washing the cells with 20/80 mixed medium (20% Roswell Park Memorial Institute (RPMI)/ 80% AIM- V medium) without serum, cells were rested for 30 min. at RT. For the trans-migration assay, 96-transwell plates were used with 3.0-μm pore polycarbonate permeable membranes (Sigma-Aldrich). Top wells contained 0.1 × 10^6^ neutrophils, and bottom wells contained 200 µL 20/80 mixed medium as a control, or 200 µL 20/80 mixed medium supplemented with a final concentration of either 100 ng Recombinant Human IL-8 (Peprotech) or 10 ng LTB-4 (Sigma-Aldrich). Additionally, 0.1 × 10^6^ neutrophils were plated in the lower well (top wells left empty) to calculate the quantity of migrated neutrophils relative to maximum migration. All conditions and controls were performed in triplicate. Plates were incubated for 40 min at 37 °C, after which the migrated neutrophils were harvested, transferred to a low-binding surface, black 96-well flat bottom OptiPlates (Perkin Elmer), and lysed using HTAB buffer (1 g/L Tween 20, 2 g/L CTAB, 2 g/L BSA and 7,44 g/L EDTA). As a readout, fluorescent Calcein was measured at excitation 485/emission 520 on a PHERAstar FS (BMG labtech) microplate reader.

### Phagocytosis assay

After isolation (as described in “Neutrophil Isolation from fresh blood samples”), neutrophils were rested for 30 min at RT in 20/80 mixed medium and transferred to 96-well plates at a concentration of 0.5 × 10^6^ cells/mL. Cells were incubated for 1 h at 37 °C with or without 50 µL/mL pHrodo™ Red *E. coli* BioParticles™ Conjugate for Phagocytosis (Thermo Fisher Scientific). All conditions and controls were performed in duplicate, of which the average was taken during the analysis. Flow cytometry was used to quantify the percentage of phagocytic neutrophils and the MFI to quantify the quantity of phagocytosis. By multiplying those two numbers, the Phagocytosis Index was calculated for each person.

### NET formation

Isolated neutrophils (as described in “Neutrophil Isolation from fresh blood samples”) were plated in DMEM medium (Thermo Fisher Scientific) at a density of 10,000 cells per well on a poly-l-lysine pre-coated eight-well Glass Bottom µ-Slide (Ibidi). Cells were allowed to adhere for 30 min at 37 °C, 5% CO_2_, before relevant wells were stimulated with 100 nM PMA. Both stimulated and unstimulated neutrophils were incubated for 4 h at 37 °C, 5% CO_2_, after which cells were washed with PBS and fixed for 30 min at RT with 2% methanol-free formaldehyde (w/v) (Thermo Fischer Scientific). Slides were stained for NETs using Abcam antibodies against myeloperoxidase (MPO) (1:50, ab11729), Citrullinated H3 rabbit (1:200, ab 150083) and goat anti-rabbit (1:500, ab5103). Subsequently, samples were mounted with ProLong™ Gold Antifade Mountant with DAPI (Thermo Fisher Scientific). Images were acquired at 20x in a blindly predefined area of the slide, on an Axio Scan (Zeiss) equipped with a Hamamatsu Orca Flash 4.0 monochrome camera. NETosis was quantified in FIJI as the fraction of neutrophils that produced a NET in a randomly selected area of fixed size.

### ROS assay

Fresh WBC were plated at a concentration of 1 × 10^6^ cells per well of a 96-well round bottom plate in the Assay Buffer that is part of the total reactive oxygen species (ROS) assay kit (Thermo Fischer Scientific). ROS staining was added to the relevant wells, and the plate was incubated for 1 h at 37 °C. As positive controls, a 100 ng/mL LPS stimulated condition and a hydrogen peroxide condition were taken along. As a negative control, cells without ROS staining were taken along, which were used to calculate the normalized MFI. After ROS staining, cells were stained for flow cytometry, and data acquisition was performed on an LSRII flow cytometer using Diva software (BD Biosciences).

### Statistical analyses

GraphPad Prism 9 software was used for statistical analysis and graphing of bar graphs of the flow cytometry and proteomics data. Kruskal–Wallis test was used when comparing more than two groups, followed by Dunn’s test to obtain adjusted *P* values. *P* values that appear in the volcano plots are corrected using the Benjamini–Hochberg procedure across immune cell populations. For two group comparisons Mann–Whitney test was applied. When testing matched samples (e.g., before and after stimulation) *p* values were computed with the Wilcoxon signed-rank test. Qlucore 3.8 software and DESeq2 in R v.4.1.0 were used for statistical analysis and graphing of the bulk RNA-sequencing data and proteomics data. ns not significant; *p < 0.05; **p < 0.01; ***p < 0.001; ****p < 0.0001.

## Supplementary information

Supplementary tables and figures

## Data Availability

Bulk RNA-sequencing data from human neutrophils in this study is deposited in GEO under accession number GSE264108. Proteomics data from neutrophils in this study have been deposited to the ProteomeXchange Consortium via the PRIDE partner repository^104^ with the identifier PXD051334.

## Code availability

All analyses were conducted using publicly accessible open-source software tools, such as DESeq2, without the development of novel code; therefore, no custom scripts are available for sharing. The DESeq2 package was run in RStudio under R version 4.1.0 for differential gene expression analysis.
