# Supplementary material for: Triple-negative breast cancer modifies the systemic immune landscape and alters neutrophil functionality
Source: NPJ Breast Cancer. 2025 Jan 23;11:5. doi: 10.1038/s41523-025-00721-2 (PMC11754814; doi:10.1038/s41523-025-00721-2)
Supplement: Supplementary file 1 — Supplementary tables and figures [file 41523_2025_721_MOESM1_ESM.pdf]

Supplementary Tables

<table>
<tr><th></th><th>Healthy donors</th><th colspan="2">Stage I-III TNBC</th><th>mTNBC Discovery cohort</th><th>mTNBC Validation cohort</th></tr>
<tr><td rowspan="3">Number of participants</td><td rowspan="3">65</td><td>Stage I</td><td>12</td><td rowspan="3">92</td><td rowspan="3">69</td></tr>
<tr><td>Stage II</td><td>27</td></tr>
<tr><td>Stage III</td><td>5</td></tr>
<tr><td>Distant metastasis</td><td>No</td><td colspan="2">No</td><td>Yes</td><td>Yes</td></tr>
<tr><td>Median age, years (range)</td><td>58 (27-73)</td><td colspan="2">50 (25-71)</td><td>55 (33-75)</td><td>51 (29-70)</td></tr>
<tr><td>Median BMI</td><td>24,5</td><td colspan="2">25,1</td><td>25,5</td><td>25,4</td></tr>
<tr><td>Previous chemotherapy exposure</td><td>x</td><td colspan="2">1 (2%)</td><td>54 (59%)</td><td>68 (99%)</td></tr>
</table>

Supplementary Table 1: basic characteristics of participating patients with TNBC and healthy donors.

**Type of prior chemotherapy**	**n**	**%**
Antracycline-taxane containing	38	70
Antracycline-taxane containing plus carboplatin	14	26
Non-antracycline containing	2	4
Adjuvant Capecitabine	23	43

Supplementary Table 2: types of chemotherapy regimens received by patients with metastatic TNBC who previously underwent chemotherapy for their primary tumor in the non-metastatic setting.

Human flow cytometry antibodies					
**Antigen**	**Fluorochrome**	**Clone**	**Dilution**	**Company**	**Catalogue number**
CD3	PE Cy5	UCHT1	1:200	BD Bioscience	555334
CD4	BV421	RPA-T4	1:100	BD Bioscience	562424
CD8	BUV805	SK1	1:200	BD Bioscience	612754
Pan γδ TCR	PE	11F2	1:100	BD Bioscience	555717
vδ1	FITC	TS8.2	1:100	Thermofisher	TCR2730
vδ2	BUV395	B6	1:100	BD Bioscience	748582
FoxP3	PE Cy5.5	FJK-16s	1:50	Thermofisher	35-5773-82
CCR7	APC R700	150503	1:50	BD Bioscience	565868
CD45RA	BUV737	HI100	1:400	BD Bioscience	612846
CD25	AF647	BC96	1:100	BioLegend	302618
PD-1	APC Cy7	EH12.2H7	1:100	BioLegend	329922
CTLA-4	PE CF594	BNI3	1:200	BD Bioscience	562742
IL-17	PerCP Cy5.5	N49-653	1:50	BD Bioscience	560799
IFNγ	BV785	4S.B3	1:200	BioLegend	502542
TNFα	PE Cy7	Mab11	1:400	BioLegend	502930
CD27	BV786	L128	1:100	BD Bioscience	563327
TIGIT	PerCP Cy5.5	A151536	1:100	BioLegend	372718
Ki-67	PE Cy7	B56	1:50	BD Bioscience	561283
CTLA-4	PE CF594	PE/Dazzle594	1:200	BioLegend	369616
CD19	PE Cy5	HIB19	1:200	BD Bioscience	555414
CD3ε	BUV496	UCHT1	1:100	BD Bioscience	612940
CD56	PE Cy5	B159	1:100	BD Bioscience	555517
CD161	PE Cy5	DX12	1:100	BD Bioscience	551138
HLA-DR	BUV661	G46-6	1:100	BD Bioscience	612980
CD14	BUV737	M5E2	1:100	BD Bioscience	612763
CD16	BUV496	3G8	1:100	BD Bioscience	612944

CD16	AF700	3G8	1:200	BioLegend	302026
CD11b	BV421	ICRF44	1:200	BioLegend	301324
CD11c	BV785	3.9	1:100	BioLegend	301644
cKIT/CD117	PE Cy5.5	104D2	1:400	Thermofisher	CD11718
CD1c	PE Cy7	L161	1:100	BioLegend	331516
CD141	BV711	1A4	1:100	BD Bioscience	563155
CD123	PE	6H6	1:200	BioLegend	396604
CD66b	PerCP-Cy5.5	G10F5	1:200	BD Bioscience	562254
CD66b	AF647	G10F5	1:200	BD Bioscience	561645
CD33	PerCP Cy5.5	WM53	1:100	BioLegend	303414
CD303	APC vio770	REA693	1:100	Miltenyi Biotech	130-114-178
CD41a	BUV395	HIP8	1:400	BD Bioscience	740295
FcεRIα	PE Dazzle 594	AER-37(CRA-1)	1:200	BioLegend	334634
CD34	FITC	581	1:100	BD Bioscience	555821
CD19	BUV395	SJ25C1	1:50	BD Bioscience	563549
IgD	APC	IA6-2	1:100	BD Bioscience	561303
CD20	BUV805	2H7	1:200	BD Bioscience	612905
CD27	PE	M-T271	1:200	BD Bioscience	555441
CD10	AF700	HI10a	1:200	BD Bioscience	563509
CD24	BB515	ML5	1:200	BD Bioscience	564521
IgM	APC Cy7	MHM-88	1:100	BioLegend	314520
CD38	BUV737	HIT2	1:400	BD Bioscience	741837
CD5	PE Dazzle 594	L17F12	1:400	BioLegend	364012
CD1d	BV786	42.1	1:200	BD Bioscience	743608
CD138	BV711	MI15	1:200	BioLegend	563184
CXCR4	PE	12G5	1:50	BioLegend	306506
CD3	PerCP Cy5.5	SK7	1:100	BioLegend	344808
CD19	PerCP Cy5.5	HIB19	1:100	BioLegend	302230

CD161	PerCP Cy5.5	HP-3610	1:100	BioLegend	339908
CD14	BV605	M5E2	1:100	BioLegend	301834
CD49d	BUV737	9F10	1:100	BD Bioscience	612850
CD62L	BUV805	DREG-56	1:100	BD Bioscience	742024
CD80	BUV395	L3074	1:100	BD Bioscience	565210
CD101	PE Cy7	BB27	1:100	BioLegend	331014
CD11b	BV785	ICRF44	1:100	BioLegend	301246
CD15	eFluor450	H198	1:100	Invitrogen	48-0159-42
CD86	BV711	IT2.2	1:100	BioLegend	305440
CD177	FITC	MEM-166	1:200	BioLegend	3115804
Siglec8	PE Dazzle 594	7C9	1:200	BioLegend	315804

Supplementary Table 3: list of antibodies used for flow cytometry.

a Myeloid panel

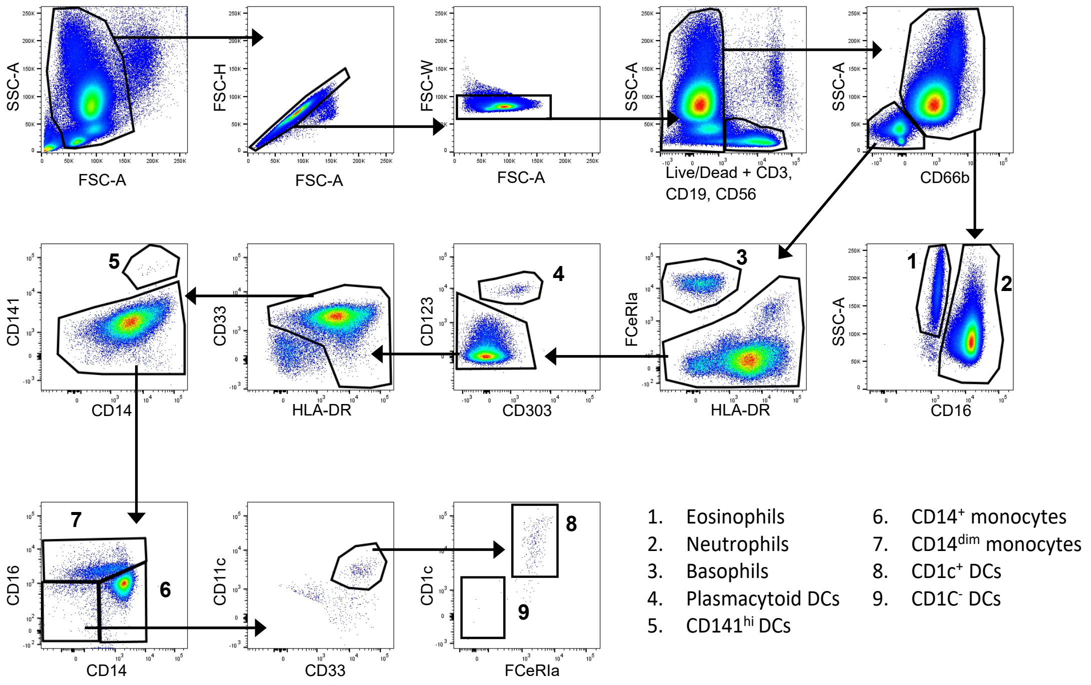

b B and NK cell panel

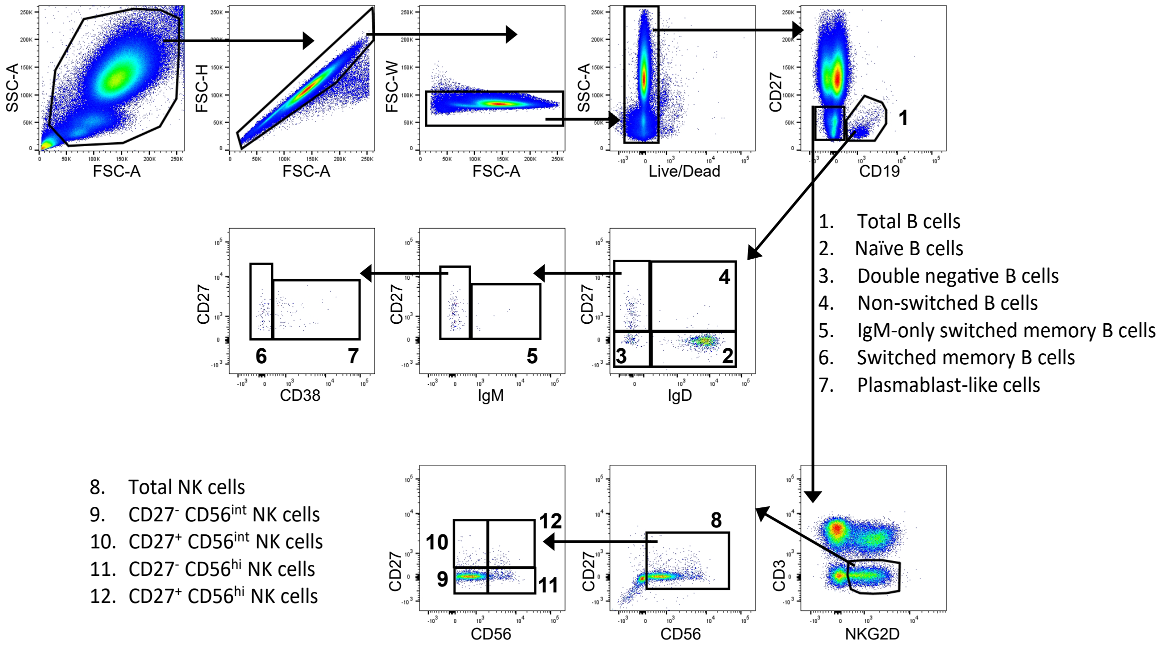

c T cell panel

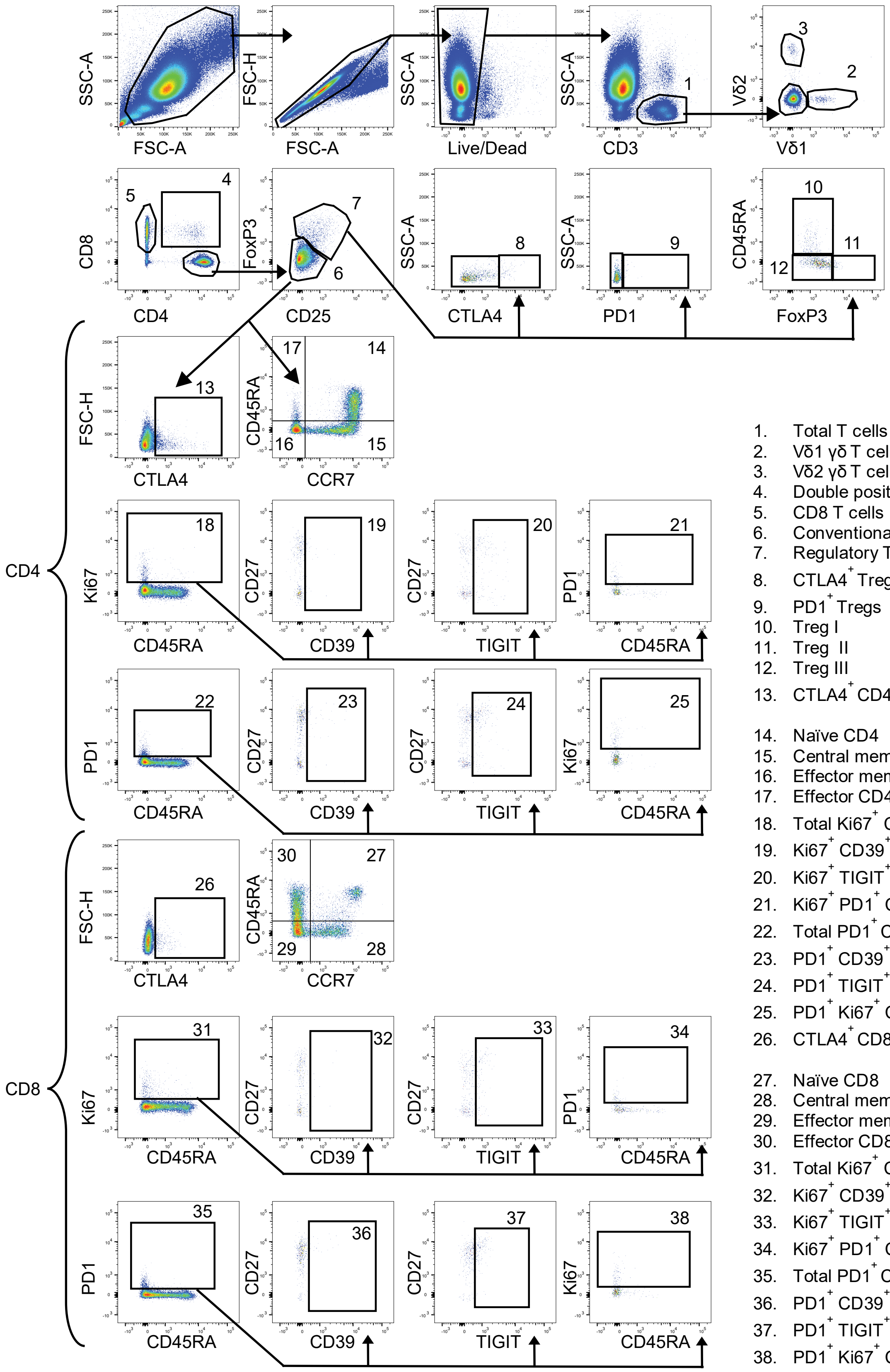

1. Total T cells
2. Vδ1 γδ T cells
3. Vδ2 γδ T cells
4. Double positive T cells
5. CD8 T cells
6. Conventional CD4 T cells
7. Regulatory T cells (Tregs)
8. CTLA4$^+$ Tregs
9. PD1$^+$ Tregs
10. Treg I
11. Treg II
12. Treg III
13. CTLA4$^+$ CD4 T cells

14. Naïve CD4
15. Central memory CD4
16. Effector memory CD4
17. Effector CD4
18. Total Ki67$^+$ CD4 T cells
19. Ki67$^+$ CD39$^+$ CD4 T cells
20. Ki67$^+$ TIGIT$^+$ CD4 T cells
21. Ki67$^+$ PD1$^+$ CD4 T cells
22. Total PD1$^+$ CD4 T cells
23. PD1$^+$ CD39$^+$ CD4 T cells
24. PD1$^+$ TIGIT$^+$ CD4 T cells
25. PD1$^+$ Ki67$^+$ CD4 T cells
26. CTLA4$^+$ CD8 T cells

27. Naïve CD8
28. Central memory CD8
29. Effector memory CD8
30. Effector CD8
31. Total Ki67$^+$ CD8 T cells
32. Ki67$^+$ CD39$^+$ CD8 T cells
33. Ki67$^+$ TIGIT$^+$ CD8 T cells
34. Ki67$^+$ PD1$^+$ CD8 T cells
35. Total PD1$^+$ CD8 T cells
36. PD1$^+$ CD39$^+$ CD8 T cells
37. PD1$^+$ TIGIT$^+$ CD8 T cells
38. PD1$^+$ Ki67$^+$ CD8 T cells

d T cell panel ~ cytokine production upon stimulation

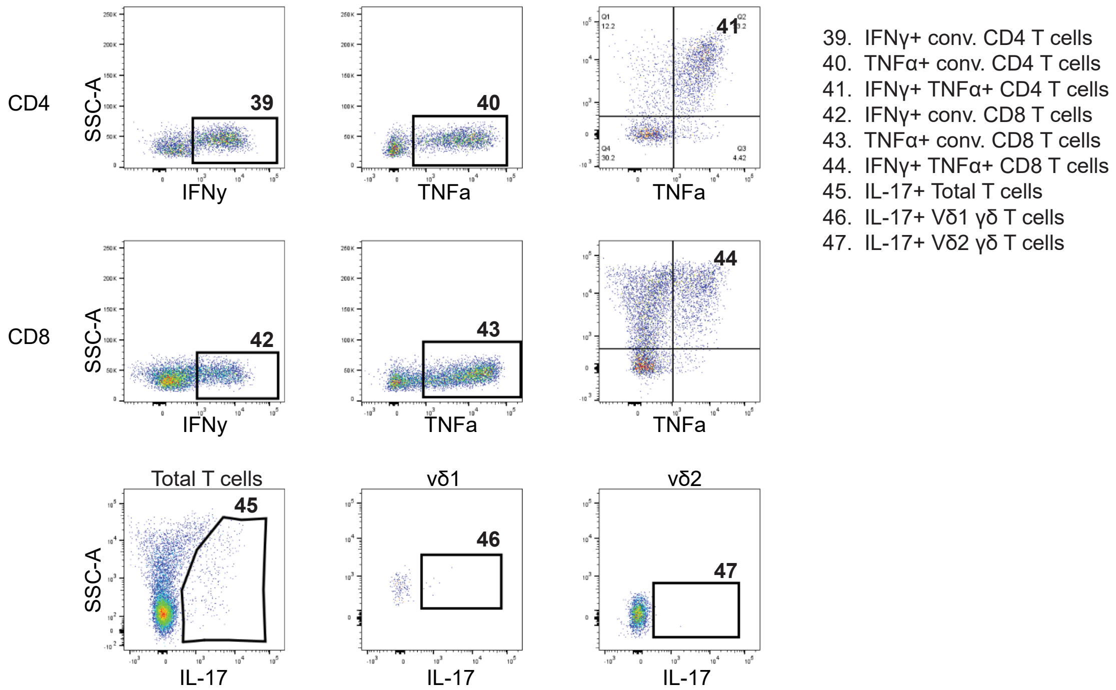

Supplementary Figure 1. Gating strategies for flow cytometry analysis of peripheral blood immune populations. (a) Myeloid panel gating strategy identifying eosinophils (lineage-, high side scatter, CD66b+ CD16-), neutrophils (lineage-, high side scatter, CD66b+ CD16+), basophils (lineage-, FcεRIα+, HLA-DR-), plasmacytoid DCs (lineage-, HLA-DR+, CD303+, CD123+), CD141hi DCs (lineage-, HLA-DR+, CD33+, CD141+), CD14+ monocytes (lineage-, HLA-DR+, CD33+, CD14+, CD16-/+), CD14dim monocytes (lineage-, HLA-DR+, CD33+, CD14dim, CD16+), CD1c+ DCs (lineage-, HLA-DR+, CD33+, CD14-, CD16-, CD1c+, FcεRIα+) and CD1c- DCs (lineage-, HLA-DR+, CD33+, CD14-, CD16-, CD1c-, FcεRIα-). **(b)** Gating strategy to identify B cell subsets identifying naive B cells (CD19+, CD27-, IgD+), double negative B cells (CD19+, CD27-, IgD-), non-switched memory B cells (CD19+, CD27+, IgD+), IgM-only switched memory B cells (CD19+, CD27+, IgD-, IgM+), switched memory B cells (CD19+, CD27+, IgD-, IgM-, CD38-/+), and plasmablasts-like cells (CD19+, CD27+, IgD-, IgM-, CD38hi). Gating strategy to identify NK cells (CD19-, CD3-, NKG2D+, CD56+), CD27- CD56int NK cells (CD19-, CD3-, NKG2D+, CD56int, CD27-), CD27+ CD56int NK cells (CD19-, CD3-, NKG2D+, CD56int, CD27+), CD27- CD56hi NK cells (CD19-, CD3-, NKG2D+, CD56hi, CD27-) and CD27+ CD56hi NK cells (CD19-, CD3-, NKG2D+, CD56hi, CD27+). **(c)** T cell panel gating strategy identifying Vδ1 γδ T cells (CD3+, Vδ1+, pan γδ TCR+), Vδ2 γδ T cells (CD3+, Vδ2+), double positive T cells (CD3+, Vδ1-, pan γδ TCR-, Vδ2-, CD8+, CD4+), CD8 T cells (CD3+, Vδ1-, pan γδ TCR-, Vδ2-, CD8+, CD4-), conventional CD4 T cells (CD3+, Vδ1-, pan γδ TCR-, Vδ2-, CD8-, CD4+, FoxP3-), Tregs (CD3+, Vδ1-, pan γδ TCR-, Vδ2-, CD8-, CD4+, FoxP3+, CD25hi), Treg I (CD3+, Vδ1-, pan γδ TCR-, Vδ2-, CD8-, CD4+, CD25hi, FoxP3int, CD45RA+), Treg II (CD3+, Vδ1-, pan γδ TCR-, Vδ2-, CD8-, CD4+, CD25hi, FoxP3hi, CD45RA-) and Treg III (CD3+, Vδ1-, pan γδ TCR-, Vδ2-, CD8-, CD4+, CD25hi, FoxP3int, CD45RA-). Differentiation states were obtained as followed for both the conventional CD4 T cells and CD8 T cells: naïve T cells (CD45RA+, CCR7+), central memory T cells (CD45RA-, CCR7+), effector memory T cells (CD45RA-, CCR7-), effector T cells (CD45RA+, CCR7-). Additional phenotypic markers were gated according to the population names. **(d)** Cytokine production was measured after PMA-ionomycin stimulation. Gating strategy identifying IFNγ+ conventional CD4 T cells (CD3+, Vδ1-, pan γδ TCR-, Vδ2-, CD8-, CD4+, FoxP3-, IFNγ+), TNFα+ conventional CD4 T cells (CD3+, Vδ1-, pan γδ TCR-, Vδ2-, CD8-, CD4+, FoxP3-, TNFα+), IFNγ+ TNFα+ conventional CD4 T cells (CD3+, Vδ1-, pan γδ TCR-, Vδ2-, CD8-, CD4+, FoxP3-, IFNγ+, TNFα+), IFNγ+ CD8 T cells (CD3+, Vδ1-, pan γδ TCR-, Vδ2-, CD8+, CD4-, IFNγ+), TNFα+ CD8 T cells (CD3+, Vδ1-, pan γδ TCR-, Vδ2-, CD8+, CD4-, TNFα+), IFNγ+ TNFα+ CD8 T cells (CD3+, Vδ1-, pan γδ TCR-, Vδ2-, CD8+, CD4-, IFNγ+, TNFα+). IL17 production is assessed by the following gatings: IL17+ total T cells (CD3+, IL17+), IL17+ Vδ1 γδ T cells (CD3+, Vδ1+, IL17+) and IL17+ Vδ2 γδ T cells (CD3+, Vδ2+, IL17+).

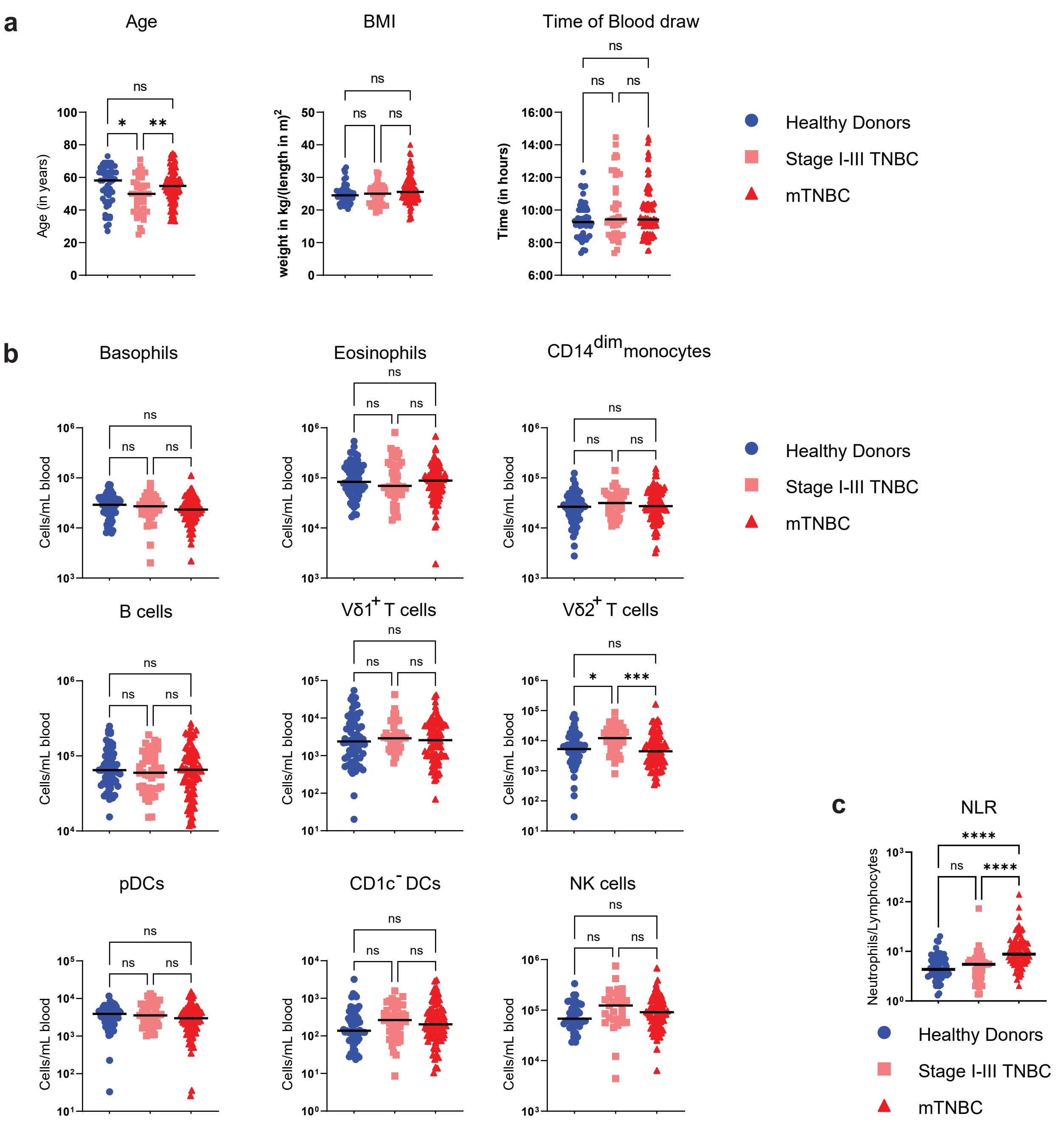

Supplementary Figure 2. Patient characteristics, NLR and immune cell subsets that did not show statistically significant abundance differences. (a) Age- and BMI-distribution and the times at which the blood was taken from the healthy donors, patients with stage I-III TNBC and patients with mTNBC that participated in our discovery cohort. **(b)** Absolute counts of major circulating immune cell subsets that were not significantly dysregulated in patients with TNBC. Depicted are cell counts per mL blood (log scale) assessed by flow cytometry in healthy donors (HDs; n=65), stage I-III (Stage I-III TNBC; n=44) and metastatic TNBC patients (mTNBC; n=92). Circulating immune cell subsets that were significantly dysregulated are depicted in main Figure 1b. **(c)** Neutrophils to Lymphocyte ratio (NLR) calculated by dividing neutrophil counts by lymphocyte counts in HDs (n=65), patients with stage I-III TNBC (n=44) and patients with mTNBC (n=92). P-values are computed with the Kruskal-Wallis test followed by Dunn's multiple comparisons correction for number of groups.

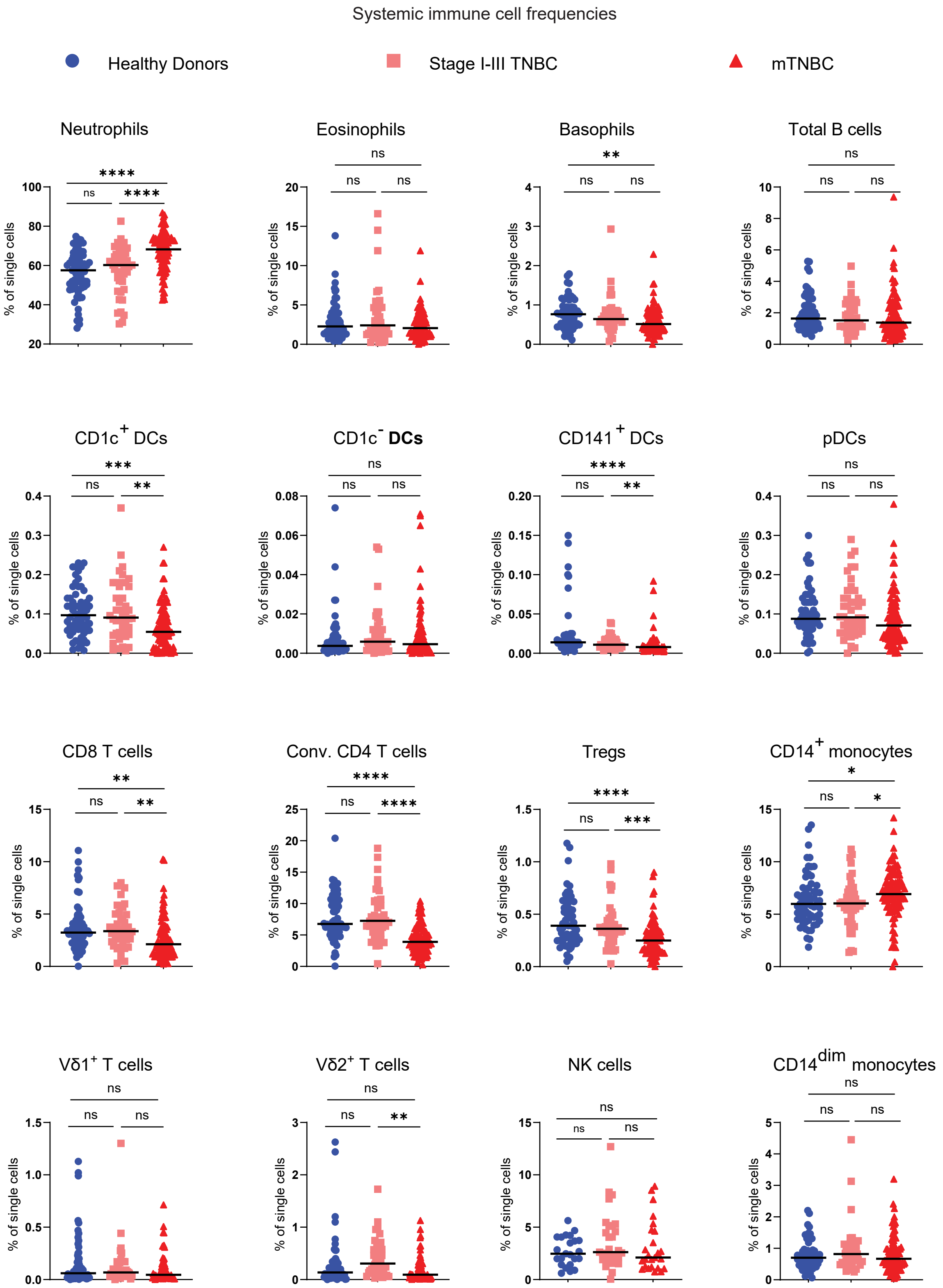

Supplementary Figure 3. Frequencies of main systemic immune cell populations. Depicted are percentages of single cells, assessed by flow cytometry in fresh blood samples from healthy donors (HDs; n=65), stage I-III (Stage I-III TNBC; n=44) and metastatic TNBC patients (mTNBC; n=92). P-values are computed with the Kruskal-Wallis test followed by Dunn's multiple comparisons correction.

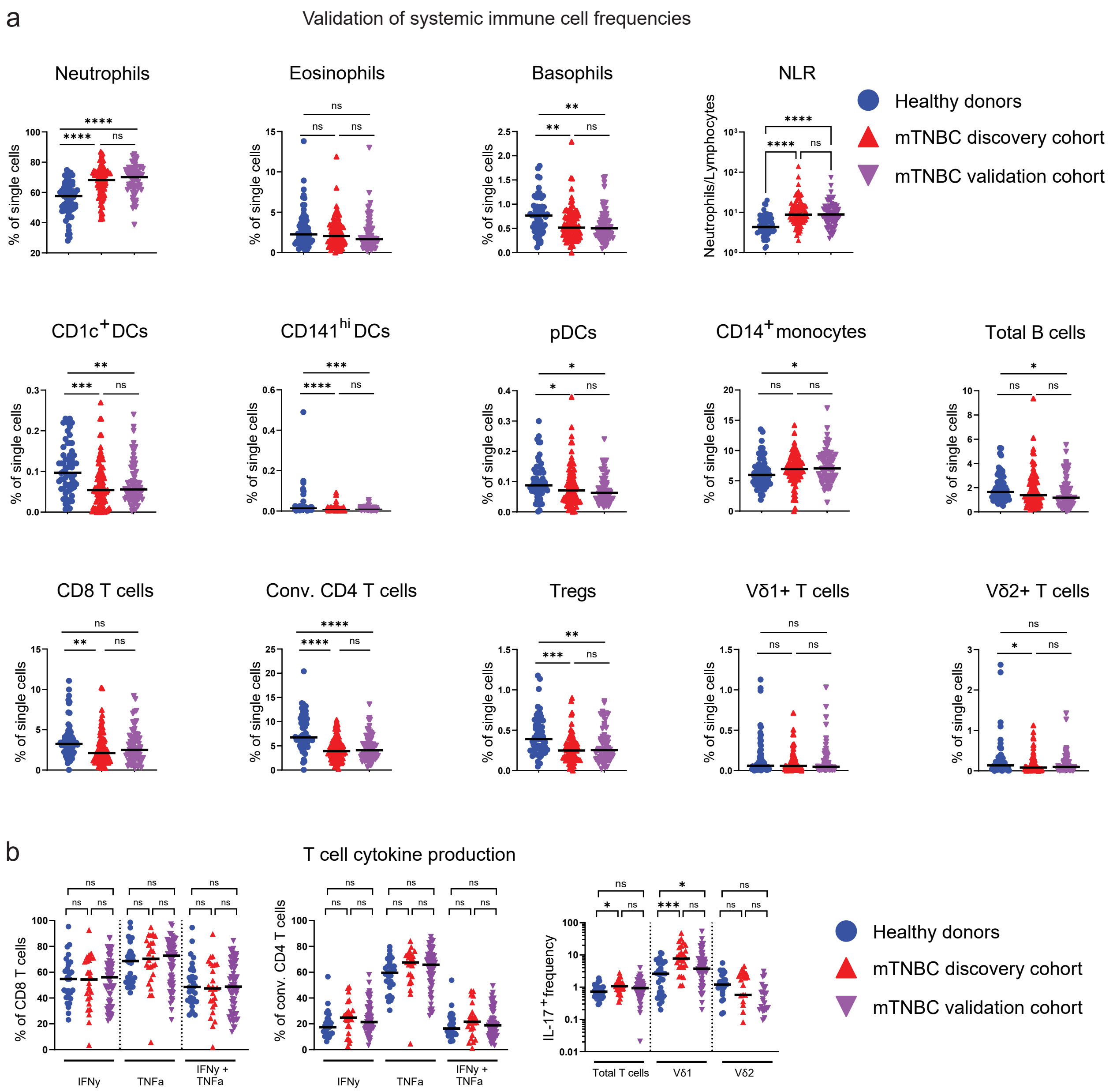

Supplementary Figure 4. Tumor induced immune perturbations to the systemic immune landscape in patients with mTNBC could be confirmed in an independent validation cohort. (a) Validation of systemic immune cell frequencies and NLR in fresh blood samples of our discovery cohort: HDs (n=65) and patients with mTNBC (n=92), and our validation cohort comprising of a group of independent patients with mTNBC (n=69). Depicted are frequencies of single cell, assessed by flow cytometry. **(b)** Validation of IFNγ and TNFα production by CD8+ and conventional CD4+ T cells, and IL17 expression on Total T cells and γδ T cells subsets Vδ1 and Vδ2 upon ex vivo stimulation, determined by flow cytometry for HDs (n=29), mTNBC patients validation cohort (n=26) and mTNBC validation cohort (n=56). P-values are computed with the Kruskal-Wallis test followed by Dunn's multiple comparisons correction.

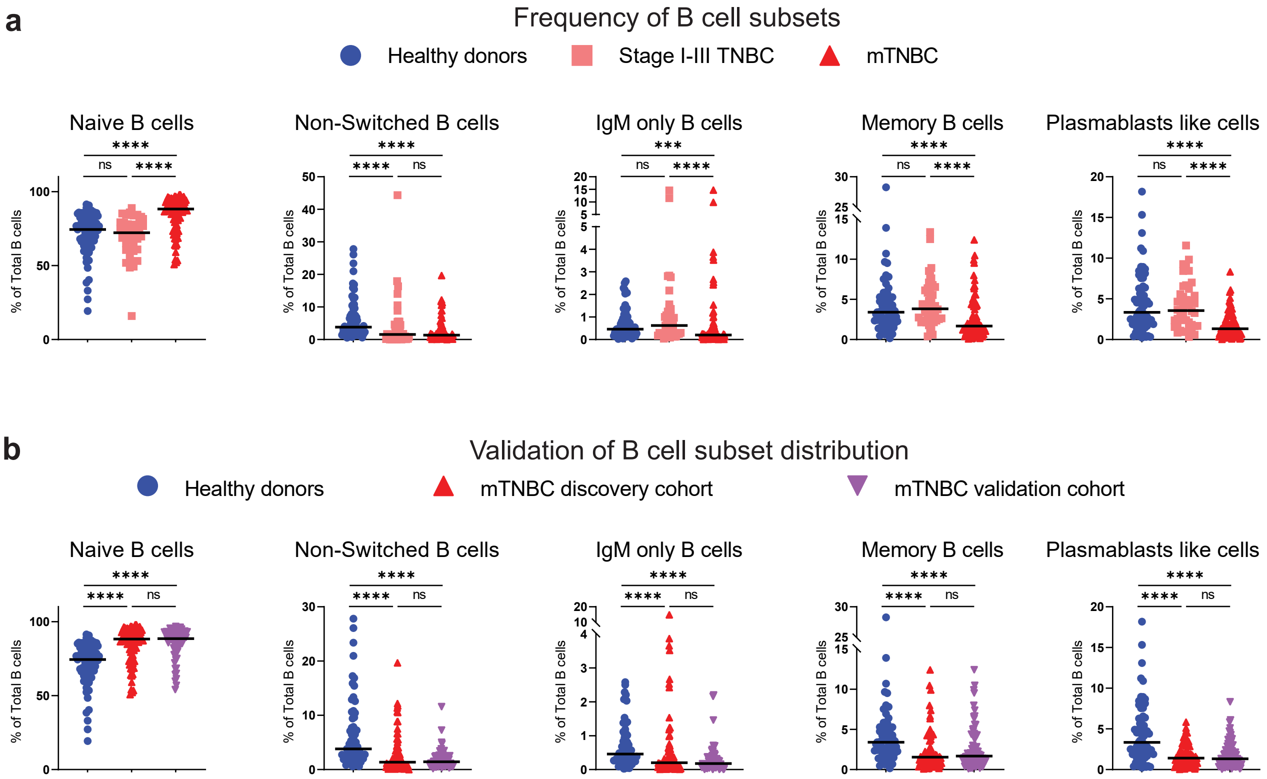

Supplementary Figure 5. Frequencies of differentiated B cell subsets. B cell subpopulations as a relative proportion of total B cells, determined by flow cytometry. **(a)** Discovery cohorts representing healthy donors (HDs; n=65), stage **I-III** (Stage **I-III** TNBC; n=44) and metastatic TNBC patients (mTNBC; n=92). **(b)** Validation cohort (mTNBC; n=69) compared to the discovery cohorts representing HDs and patients with mTNBC described in a. P-values are computed with the Kruskal-Wallis test followed by Dunn's multiple comparisons correction.

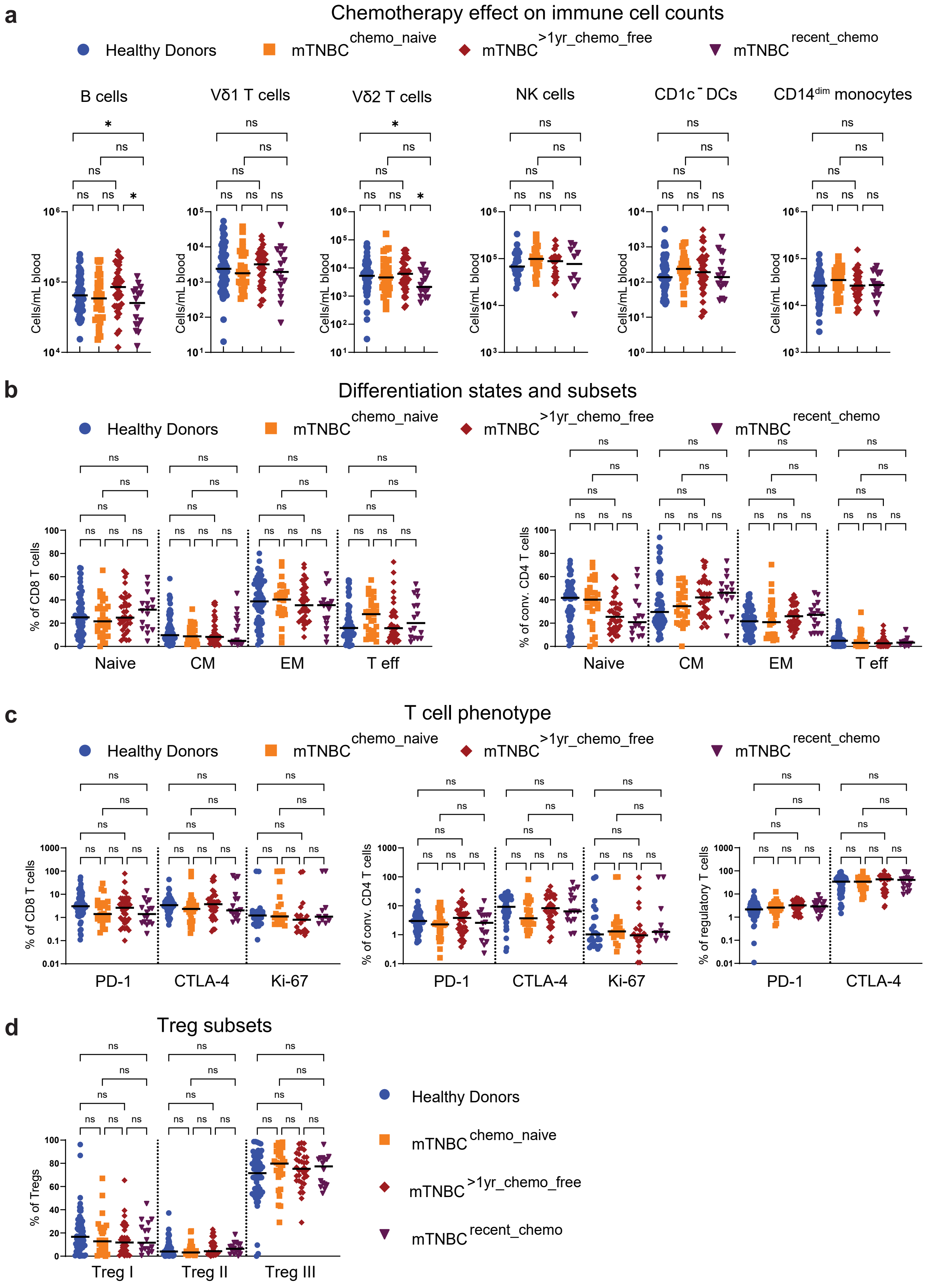

Supplementary Figure 6. Prior chemotherapy treatment does not significantly impact T cell differentiation state and phenotype in patients with metastatic triple-negative breast cancer.
(a) Prior chemotherapy effect on circulating immune cell populations that were not significantly dysregulated in patients with TNBC according to main Figures 1c and 4b-d.

(b) Differentiation state of CD8+ T cells and conventional CD4+ T cells, based on surface marker expression of CD45RA and CCR7 determined by flow cytometry and grouped based on prior chemotherapy. HD (n=65), mTNBCchemo_naïve (n=29), mTNBC>1yr_chemo_free (n=38) and mTNBCrecent_chemo (n=16). CM = central memory, EM = effector memory and T eff = effector T cells. **(c)** T cell phenotype as determined by flow cytometry comparing fractions within CD8+, conventional CD4+, and regulatory T cells for HD (n=65), mTNBCchemo_naïve (n=29), mTNBC>1yr_chemo_free (n=38) and mTNBCrecent_chemo (n=16). **(d)** Regulatory T cell subset distribution based on relative expression of FoxP3 and CD45RA as determined by flow cytometry. All p-values are computed with the Kruskal-Wallis test followed by Dunn's multiple comparisons test.

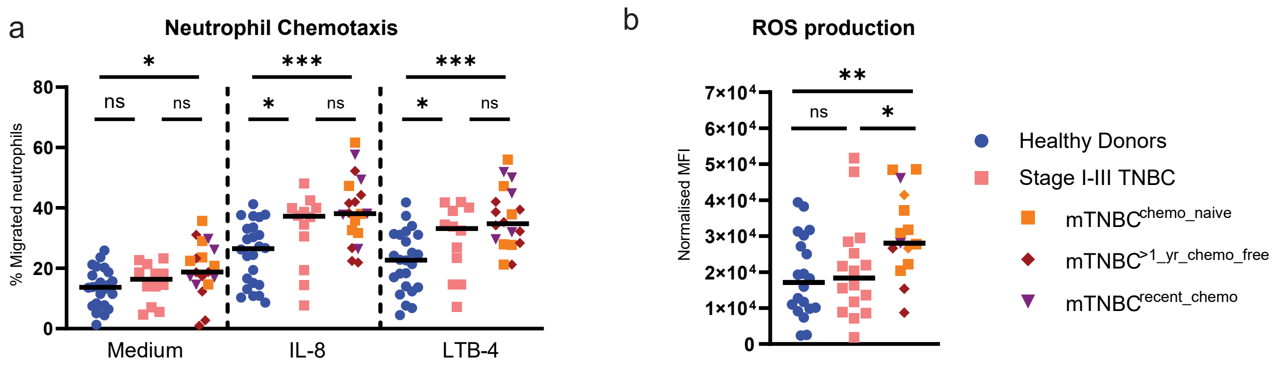

Supplementary Figure 7. Increased migration and ROS production by neutrophils from patients with metastatic triple-negative breast cancer compared to healthy donors seems unaffected by prior chemotherapy treatment. (a) Neutrophil migration rates using Medium alone, IL-8 and LTB-4 as chemo-attractants and fresh neutrophils from HDs (n=24), stage **I-III** TNBC patients (n=12) and mTNBC patients (n=20), as shown in main figure 5, but now patients with mTNBC are colored based on prior chemotherapy treatment group. P-values are computed with the Mann-Whitney U-test. **(b)** Reactive Oxygen Species (ROS) production by neutrophils isolated from fresh blood samples of HDs (n=20), patients with stage **I-III** TNBC (n=15) and patients with mTNBC (n=15), as shown in main Figure 6, but now patients with mTNBC are colored based on prior chemotherapy treatment group. P-values are computed with the Mann-Whitney U-test.
